# Genetic Basis for *Saccharomyces cerevisiae* Biofilm in Liquid Medium

**DOI:** 10.1534/g3.114.010892

**Published:** 2014-07-09

**Authors:** Kaj Scherz Andersen, Rasmus Bojsen, Laura Gro Rejkjær Sørensen, Martin Weiss Nielsen, Michael Lisby, Anders Folkesson, Birgitte Regenberg

**Affiliations:** * Department of Biology, University of Copenhagen, Copenhagen, Denmark; † Department of Systems Biology, Technical University of Denmark, Copenhagen, Denmark

**Keywords:** biofilm, PKA, adhesion, genome-wide screen, multicellular

## Abstract

Biofilm-forming microorganisms switch between two forms: free-living planktonic and sessile multicellular. Sessile communities of yeast biofilms in liquid medium provide a primitive example of multicellularity and are clinically important because biofilms tend to have other growth characteristics than free-living cells. We investigated the genetic basis for yeast, *Saccharomyces cerevisiae*, biofilm on solid surfaces in liquid medium by screening a comprehensive deletion mutant collection in the Σ1278b background and found 71 genes that were essential for biofilm development. Quantitative northern blots further revealed that *AIM1*, *ASG1*, *AVT1*, *DRN1*, *ELP4*, *FLO8*, *FMP10*, *HMT1*, *KAR5*, *MIT1*, *MRPL32*, *MSS11*, *NCP1*, *NPR1*, *PEP5*, *PEX25*, *RIM8*, *RIM101*, *RGT1*, *SNF8*, *SPC2*, *STB6*, *STP22*, *TEC1*, *VID24*, *VPS20*, *VTC3*, YBL029W, YBL029C-A, YFL054C, YGR161W-C, YIL014C-A, YIR024C, YKL151C, YNL200C, YOR034C-A, and YOR223W controlled biofilm through *FLO11* induction. Almost all deletion mutants that were unable to form biofilms in liquid medium also lost the ability to form surface-spreading biofilm colonies (mats) on agar and 69% also lost the ability to grow invasively. The protein kinase A isoform Tpk3p functioned specifically in biofilm and mat formation. In a *tpk3* mutant, transcription of *FLO11* was induced three-fold compared with wild-type, but biofilm development and cell–cell adhesion was absent, suggesting that Tpk3p regulates *FLO11* positive posttranscriptionally and negative transcriptionally.

The study provides a resource of biofilm-influencing genes for additional research on biofilm development and suggests that the regulation of *FLO11* is more complex than previously anticipated.

Many microorganisms have the ability to form the multicellular, sessile, surface-bound communities known as biofilms. Biofilm formation has been described in prokaryotes such as the Gram-negative *Pseudomonas aeruginosa* and the eukaryotic yeasts *S. cerevisiae*, *Candida albicans*, and *Candida glabrata* (Hawser and Douglas 1994; Reynolds and Fink 2001; Hall-Stoodley *et al.* 2004). Cells in biofilms are reported to have a higher degree of diversity, so they have more possible fates than cells in free-living planktonic form. One consequence of this diversity is the high number of antibiotic-persistent cells in *P. aeruginosa* biofilms, a result of the high frequency of slow-growing or dormant cells in mature biofilms (Nguyen *et al.* 2011).

Although the molecular basis for biofilm development and biofilm cell diversification has been studied extensively in bacteria, less is known about the genetic basis for biofilm formation and cell diversification in eukaryotic microbes such as yeasts. Laboratory *S*. *cerevisiae* strains have, in some cases, been selected to not form biofilms (Liu *et al.* 1996). The trait can reappear in suppressor mutants that derepress expression of the cell wall protein Flo11p or other members of the Flo protein family that induce cell–cell adhesion (Fichtner *et al.* 2007; Torbensen *et al.* 2012). The *S. cerevisiae* strain Σ1278b naturally forms biofilm in liquid medium on solid surfaces such as polystyrenes because it expresses Flo11p (Reynolds and Fink 2001). In addition to its importance for biofilm formation, Flo11p is also essential for other morphotypes, including haploid-invasive growth on complex solid medium and diploid-pseudohyphal growth (Lo and Dranginis 1998). A fourth Flo11p-dependent phenotype is a type of giant colony that develops on semisolid complex mediums at room temperature. The giant colonies have been denoted surface-spreading biofilm as well as mats in the literature (Reynolds and Fink, 2001; Ryan *et al.* 2012). Although biofilm in liquid medium, surface-spreading biofilm (mats), invasive, and pseudohyphal growth are dependent on *FLO11*, they cannot *a priory* be expected to be regulated in identical fashions because the growth conditions required for induction of these phenotypes are different.

The *FLO11* gene is located in the middle of the right arm of chromosome IX (Lo and Dranginis 1996) and has a 2.8-kb promoter (Rupp *et al.* 1999). The relatively large promoter contains an extensive set of *cis*-acting elements that respond to multiple signaling pathways (Brückner and Mösch 2011). *FLO11* is regulated by a mitogen-activated protein kinase (MAPK) pathway via the Ste12p/Tec1p transcription factors (Roberts and Fink 1994; Köhler *et al.* 2002; Rupp *et al.* 1999). The pH-sensitive Rim101p pathway regulates *FLO11* (Barrales *et al.* 2008; Bayly *et al.* 2005; Lamb and Mitchell 2003), and the response is believed to include components from the endosomal sorting complex required for transport (ESCRT), because ESCRT I, II, and III proteins are required for activation of Rim101p and transcription of *FLO11* (Sarode *et al.* 2011; Xu *et al.* 2004). Nutrient levels regulate *FLO11* transcription through other pathways. Amino acid levels influence *FLO11* transcription via the general control nonderepressible (GCN) pathway (Braus *et al.* 2003), which induces transcription on amino acid starvation (Lucchini *et al.* 1984). The presence of amino acids induces the Ssy1p-Ptr3p-Ssy5p-sensor complex, which regulates *FLO11* transcription through amino acid permeases (Torbensen *et al.* 2012). Furthermore, glucose depletion induces *FLO11* via the AMP kinase homolog Snf1p by inactivating the transcriptional repressors Nrg1p and Nrg2p (Kuchin *et al.* 2002; Van De Velde and Thevelein 2008). Low glucose is also known to induces transcription of *FLO11* through G-protein-coupled glucose receptor Gpr1p, cAMP (Van De Velde and Thevelein 2008), the protein kinase A (PKA) isoform Tpk2p, and the competing transcription regulators Sfl1p and Flo8p (Robertson and Fink 1998; Rupp *et al.* 1999). *FLO11* is repressed when Sfl1p is bound and a noncoding RNA is transcribed in the *FLO11* promoter and in a transcriptionally permissive state when Flo8p is bound to the *FLO11* promoter and the ncRNA gene is transcriptionally inactive (Bumgarner *et al.* 2009). Flo8p activity is thought to facilitate the binding of other positive transcription factors such as Tec1p, Ste12p, and Pol II that reinforce the active state of the *FLO11* promoter (Bumgarner *et al.* 2012). An interesting aspect of *FLO11* regulation is the toggle switch that results from competition between Sfl1p and Flo8p and leads to variegated *FLO11* expression (Bumgarner *et al.* 2009, 2012). Because of variable expression, only a subpopulation of cells expresses *FLO11* and contributes to cell–cell adhesion. Variegated *FLO11* expression is seen in pseudohyphal and invasive growth and could play a role in development of biofilm in liquid medium.

In contrast to *FLO11* induction through active Tpk2p, the PKA Tpk3p is reported to repress *FLO11*. This has been shown with *tpk3* mutants that have three-fold higher levels of *FLO11* mRNA than wild-type *TPK3* cells and show more robust invasive and pseudohyphal growth (Robertson and Fink 1998). Because *FLO11* expression is repressed in a *tpk3tpk2* double mutant, Tpk3p is thought to inhibit Tpk2p activity (Robertson and Fink 1998).

*FLO11* regulation has mainly been investigated under conditions in which cells grow invasively or form mats or pseudohyphae (Brückner and Mösch 2011). Because the growth conditions essential for biofilm in liquid medium are very different from those favoring mats and pseudohyphal and invasive growth, it is unknown if the transcriptional program that regulates biofilm also regulates the other *FLO11*-dependent phenotypes. On abiotic surfaces, biofilms are formed in synthetic media with glucose as the carbon source (Torbensen *et al.* 2012; Reynolds and Fink 2001), whereas surface-spreading biofilm formation and invasive growth both occur on complex solid medium, and pseudohyphal growth is formed by diploid cells on solid, synthetic, nitrogen-poor medium (Reynolds and Fink 2001; Gimeno *et al.* 1992; Roberts and Fink 1994). A recent screen for genes essential for mat formation and invasive and pseudohyphal growth found limited overlap between genes regulating the three phenotypes (Ryan *et al.* 2012), suggesting a dedicated transcriptional program for *FLO11*-dependent biofilm formation. In addition to *FLO11*, other *FLO* genes and conditions might influence biofilms, including genes regulating the extracellular matrix that strengthens the three-dimensional structure of biofilms (Kuthan *et al.* 2003; Vachova *et al.* 2011; Guo *et al.* 2000), and quorum signaling that might coordinate the developmental program. Quorum signaling is reported for yeast, but its involvement in biofilm development is unknown (Chen and Fink 2006; Smukalla *et al.* 2008; Palkova *et al.* 1997).

In the current work, we investigated the molecular basis for Σ1278b biofilm development, including the extent to which biofilm development was dependent on *FLO11*. To do this, we screened a global collection of deletion mutants in the Σ1278b background for biofilm-forming ability on a solid abiotic surface. The extent to which the molecular program for biofilm formation overlapped with other *FLO11*-dependent phenotypes was investigated by testing biofilm-deficient mutants for ability to form mats and grow invasively. We found that a substantial fraction of Σ1278b cells were not part of the biofilm but existed as planktonic cells. Genes involved in the planktonic phenotype were identified by screening for Σ1278b mutants with more biofilm-phenotype cells in the total cell mass. Our study gives comprehensive insight into the molecular program controlling biofilm development in the genetic tractable yeast *S. cerevisiae*.

## Materials and Methods

### Strains

*S. cerevisiae* Σ1278b YS-11 (*MAT***a** *can1Δ*::*STE2p-spHIS5 lyp1Δ*::*STE3p-LEU2 his3*::*HisG leu2Δ ura3Δ*) was used as a reference strain (Boone Lab, University of Toronto) (Ryan *et al.* 2012). The 4019 deletion mutants of Σ1278b YS-11 were from Ryan *et al.* (2012) and have the same barcodes and deletions as the S288c collection (Giaever *et al.* 2002).

### Media

Synthetic complete (SC) media for biofilm formation on polystyrene were made as previously described (Guthrie and Fink 1991), with the exception that amino acids and nucleotides were added in the following concentrations: adenine sulfate 20 mg/liter; uracil 38 mg/liter; l-histidine 38 mg/liter; l-arginine 38 mg/liter; l-tryptophan 38 mg/liter; l-methoinine 38 mg/liter; l-tyrosine 15 mg/liter; l-leucine 57 mg/liter; l-isoleucine 57 mg/liter; l-lysine 57 mg/liter; l-phenylalanine 48 mg/liter; l-valine 57 mg/liter; and l-threonine 57 mg/liter. Yeast extract peptone dextrose (YPD) complex medium was made as described (Guthrie and Fink 1991) using 20 g/liter agar for invasive growth and 3 g/liter for mats.

### Assay for biofilm

Precultures were grown overnight at 30° in synthetic medium with 0.2% glucose and 100 mM NH_4_^+^. Cells were subsequently inoculated into synthetic medium to OD_600nm_ 0.1 for 2 hr before biofilm assays. Assays were in 200 µL in flat-well polystyrene microtiter plates (Frisenette). Cell density of the total population or of biofilm or planktonic subpopulations was recorded at OD_450nm_ at indicated time points. Planktonic subpopulations were measured by removing nonadhering cells by pipetting and measuring cell density in new microtiter wells. Biofilm subpopulations were measured by addition of 200 µL fresh medium to adhering cells and measuring OD_450nm_. To visualize biofilms, crystal violet (HT901-8FOZ; Sigma-Aldrich) was added to wells for 24 hr at a final concentration of 0.05%. Planktonic cells and medium were removed and wells were washed four times with 200 µL H_2_O. Biofilms were dried and resuspended in 170 µL 96% ethanol for 1 hr. Biomass was determined at OD_595nm_.

### Biofilm screens of the deletion strain collection

Deletion mutants were grown on solid YPD for 2 d. Cells were subsequently transferred to 96-well flat-bottom microtiter plates (Frisenette) containing 200 µL SC medium with 0.2% glucose. Cells were propagated for 46 hr or 96 hr at 30° and biomass was determined at OD_600nm_ using a Synergy ^1^H Hybrid Reader (Biotek). All experiments were performed in triplicate. Crystal violet staining was as mentioned except that washing and OD_595nm_ measurements used a Biomek 2000 robot.

### Data analysis

Crystal violet biofilm measurements for Σ1278b YS-11 mutants were normalized to the total biomass and log-transformed as ln(OD_595nm_/OD_600nm_). Normalized biofilm scores were used to determine the median biofilm score for each mutant for both 46 hr and 96 hr. Median values were used for all further analysis (Supporting Information, File S5 and File S6). Samples with a total biomass less than OD_600nm_ <0.01 were excluded from analysis. Replicate biofilm assays of the parental YS-11 strain (n = 288) showed normalized biofilm values that followed a Gaussian distribution at both 46 hr and 96 hr. The average normalized biofilm value ±2σ of the parental strain was used to determine mutants that had a significantly different biofilm score at 46 hr and 96 hr. Mutants with median-normalized biofilm scores less than 0.584 were considered to form significantly less biofilm than the parental strain and mutant with scores more than 1.972 were considered to form significantly more biofilm than the parental strain.

### Synthetic genetic array

Selection of *tpk3 geneX* double mutants in the parental (*MAT***a** *can1Δ*::*STE2p-spHIS5 lyp1Δ*::*STE3p-LEU2 his3*::*HisG leu2Δ ura3Δ*) background was essentially conducted as described previously (Tong *et al.* 2001).

### Confocal laser scanning microscopy

Overnight cultures were diluted to OD_600nm_ 0.1 in synthetic complete medium (0.2% glucose) and incubated on Rinzl plastic coverslips (Electron Mictoscopy Sciences) for 96 hr at 30°. Biofilms were washed twice in saline before 30 min of FUN-1 (Invitrogen) staining. Imaging used a plan-Neofluar 40×/1.3 oil microscopy objective with a Zeiss LSM510 microscope.

### RNA FISH

RNA FISH was conducted essential as described previously (McIsaac *et al.* 2013) using Stellaris probes from Biosearch Technologies. Thirty-five 20mer Quasar 670 probes covering the stretch +9 to +708 of *FLO11* were used for detection of *FLO11* mRNA, whereas 35 20mer Quasar 570 probes covering the stretch +2 to +701 of *ACT1* were used as positive hybridization control. Precultures were grown overnight at 30° in synthetic medium. Cells were subsequently inoculated into synthetic medium to OD_600nm_ 0.1 for 20 hr before fixation. Cells were fixated with 3% paraformaldehyde for 30 min at 30° followed by 4-hr incubation at 5°. Expression of *FLO11* was subsequently recorded with a Zeiss LSM780 microscope. Cells were counted as recordable if they were labeled with the *ACT1* probe. All *ACT1* positive cells were subsequently recorded for their expression of *FLO11* mRNA by counting cells with one or more red foci as positive for *FLO11* mRNA. A total of 1329 wild-type cells, 1167 *sfl1* cells, and 542 *flo11* cells were counted as blinded samples. The *flo11* cells served a negative control for *FLO11* mRNA expression. None of the *flo11* cells showed any signal for *FLO11* mRNA labeling.

### Glucose concentration

Glucose concentration was determined enzymatically with a glucose assay kit (GAGO-20; Sigma-Aldrich) adjusted for use with microtiter plates.

### Invasive growth

Invasive growth was assayed essentially as described previously (Roberts and Fink 1994) Cells were patched on solid 2.0% agar YPD using an inoculation loop and propagated for 3 d at 30°. Plates were covered with water for 30 min and shaken carefully, and nonadhering cells were removed. Complete colonies or colonies with portions remaining in or on the agar were categorized as invasive.

### Mat formation

Cells were patched in the center of 25 ml YPD with 0.3% agar and incubated for 5 d at room temperature (22°–25°). Colonies with a structured hub and spokes were categorized as mat formers. Colonies that were completely smooth were categorized as having lost the ability to form mats.

### Northern blot

RNA for northern dot blots was purified from mutants and the parental strain, YS-11, grown for 96 hr in square 120-mm × 120-mm Petri dishes (Frisenette). Total RNA was purified as previously described (Torbensen *et al.* 2012). All samples were treated with DNAse and tested for removal of DNA by PCR using *ACT1* primers 5′-TGGATTCTGGTATGTTCTAGC-3′ and 5′-GAACGACGTGAGTAACACC-3′. Samples that still contained DNA were treated further with DNAse until no trace of DNA was detected by PCR. DNAse-treated RNA, 2 µg in 3 µl, was dropped onto Hydrobond-N^+^ membranes (GE Healthcare) and dried. Membranes were wrapped in plastic wrap and RNA was cross-linked by UV light for 30 sec at 302 nm. One set of membranes was hybridized to a *FLO11* [^32^P]-labeled probe and another set was hybridized to a [^32^P]-labeled *ACT1* probe. Hybridization and probes were as described (Torbensen *et al.* 2012). Hybridization to each dot was recorded with a Storm 840 phosphorimager (Bio-Rad), and Molecular Dynamics ImageQuant TL software was used for quantification. *FLO11* transcript levels were normalized to *ACT1* transcript levels and the average normalized *FLO11* transcript level was calculated from three independent experiments for each mutant and parental strain.

## Results

### *S. cerevisiae* Σ1278b formation of mixed biofilm and planktonic populations is *FLO11*-dependent

**Figure 1:**
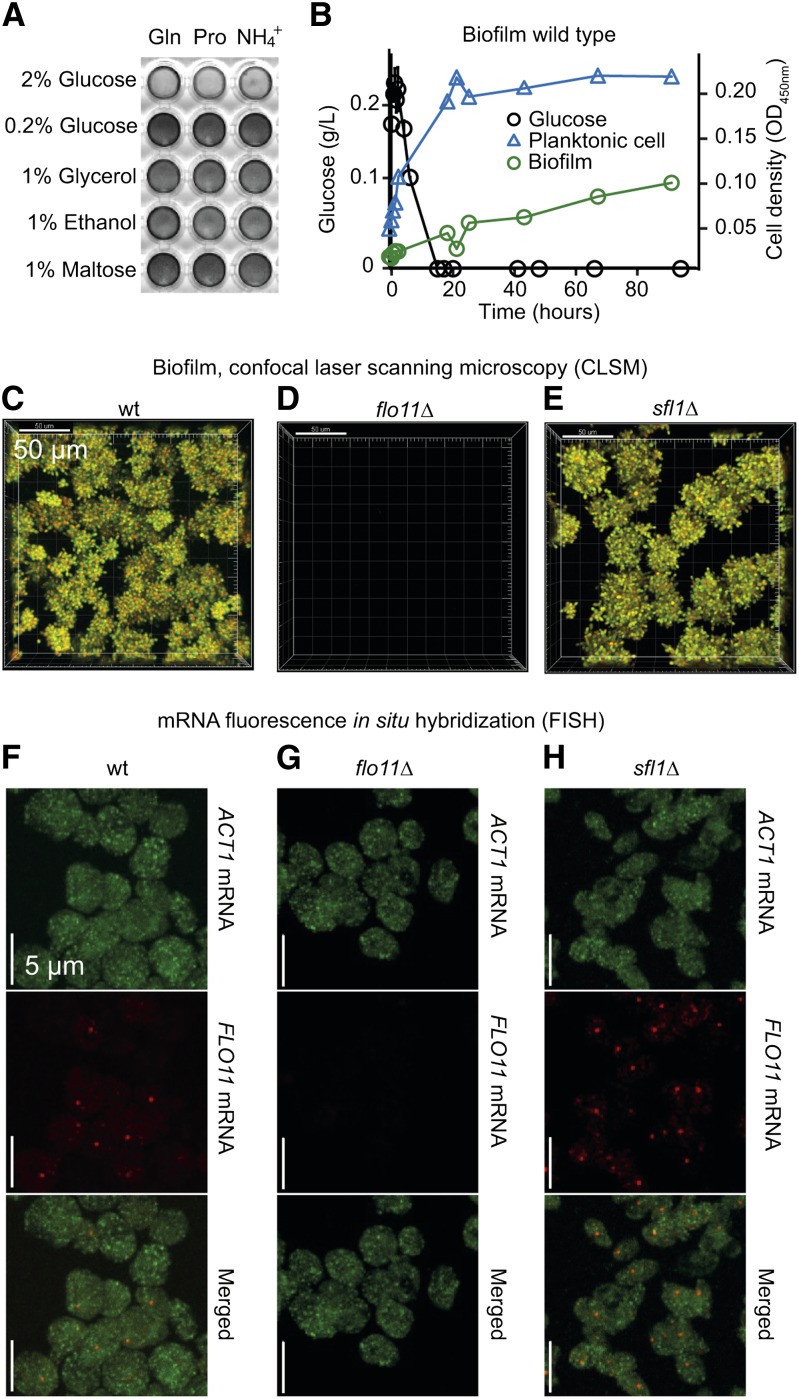
*S. cerevisiae* Σ1278b forms mixed populations of biofilm and planktonic cells. (A) Wild-type cells grown at 30° in polystyrene wells in synthetic complete (SC) media in indicated carbon and nitrogen sources (Gln = glutamine 100 mM; Pro = proline 100 mM; NH_4_^+^ = ammonium 100 mM) for 96 hr and stained with crystal violet. Dark staining indicates biofilm formation. (B) Biofilm formation on polystyrene by a wild-type (wt) population in SC 0.2% glucose and NH_4_^+^. Blue triangles = nonadhering planktonic cells; green circles = biofilm-forming cells. Planktonic cells were separated from biofilms by pipetting. Cell density was measured at OD_450nm_. Black circles = measured glucose concentration left in growth medium over time. (C, D, E) Biofilm was recorded with confocal laser scanning microscopy (CLSM) after 96 hr of growth in SC 0.2% glucose and NH_4_^+^. Nonadhering cells were removed by a single gentle pipetting and adhering cells were dyed with FUN-1. White bar = 50 µm. (F, G, H) Single cell fluorescence *in situ* hybridization (FISH) of representative samples of cells grown in SC 0.2% glucose and NH_4_^+^ for 20 hr. Bars = 5 µm. (C, F) Wild-type. (D, G) *flo11*::*kanMX* in the wild-type background. (E, H) *sfl1*::*kanMX* in the wild-type background; *MAT***a** *can1Δ*::*STE2p-spHIS5 lyp1Δ*::*STE3p-LEU2 his3*::*HisG leu2Δ ura3Δ*.

In low glucose, haploid *S. cerevisiae* Σ1278b forms biofilm on polystyrene (Reynolds and Fink 2001). We tested this phenotype in liquid synthetic medium with different carbon sources and found that haploid Σ1278b formed a biofilm when cultured in 0.2% glucose, 1% maltose, 1% glycerol, or 1% ethanol, whereas biofilm formation was repressed in 2% glucose (Figure 1A). Further examination of biofilms formed in 0.2% glucose revealed that only 25% of cells were part of the biofilm (Figure 1B). Confocal laser scanning microscopy (CLSM) revealed that the proportion of biofilm-forming cells decreased substantially in a *flo11* mutant, confirming that *FLO11* was responsible for biofilm formation (Figure 1, C and D). Fluorescent *in situ* hybridization (FISH) revealed that only a minor fraction of reference cells expressed *FLO11* mRNA (21%, n = 1329) in the synthetic 0.2% glucose medium (Figure 1F) corresponding to the fraction of cells participating in biofilm (Figure 1B). The proportion of cells expressing *FLO11* mRNA increased to 73% (n = 1167) in a *sfl1* mutant (Figure 1H), suggesting that Sfl1p took part in participation of cells in a planktonic and a biofilm-forming subpopulation (Figure 1B). This hypothesis was supported by CLSM of biofilm formed by *sfl1* and the reference strain (Figure 1, C and E). Both strains formed microcolonies, and microcolonies formed by the *sfl1* mutant appeared to be larger than those of the reference strain (Figure 1, C and E and Figure S1). Based on these initial experiments, we expected that other molecular factors involved in biofilm development and repression of biofilm could be found.

### Screening of a Σ1278b deletion collection for genes essential for biofilm development

To identify genes essential for biofilm in liquid medium in Σ1278b, as well as genes that regulate the proportion of biofilm-forming cells in a population, we screened a complete library of Σ1278b haploid mutants deleted in 4019 nonessential genes for biofilm-forming ability. The mutants were constructed by substituting each open reading frame (ORF) in Σ1278b (*MAT***a** *can1Δ*::*STE2p-spHIS5 lyp1Δ*::*STE3p-LEU2 his3*::*HisG leu2Δ ura3Δ*) with a *kanMX* cassette derived from the S288c deletion collection (Ryan *et al.* 2012). Thus, mutant alleles in the Σ1278b mutant collection were identical to the mutant alleles in the S288c collection (Giaever *et al.* 2002).

**Figure 2:**
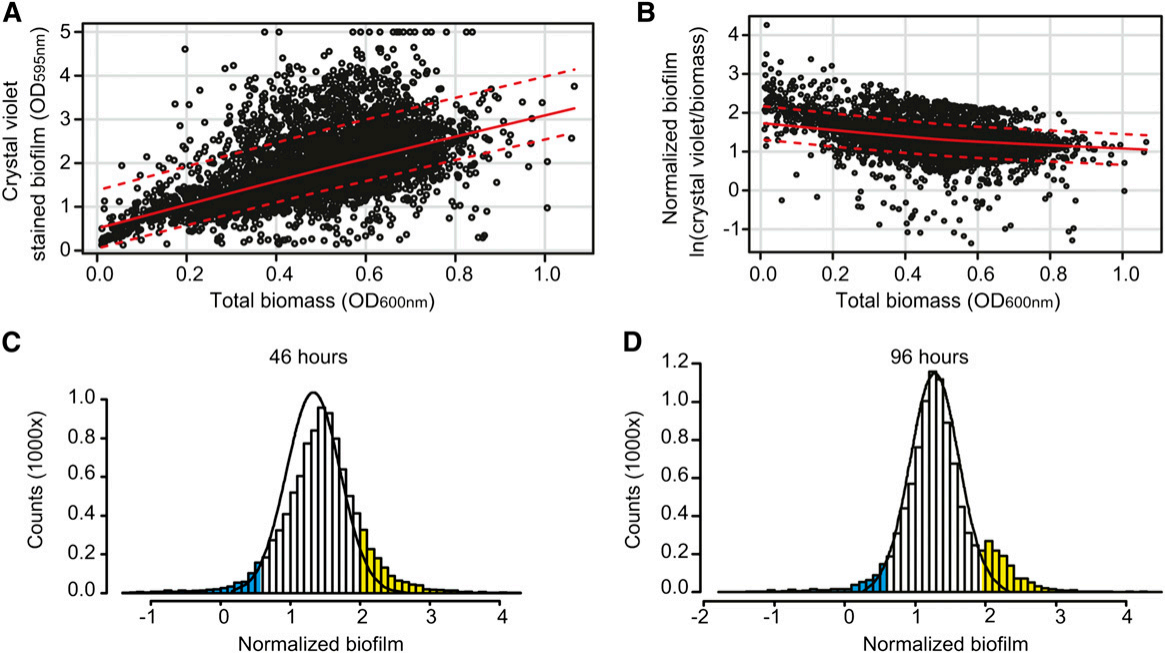
Screening of a Σ1278b deletion mutant collection for genes essential in biofilm development. Mutants deleted for one of 4019 nonessential genes (indicated as *geneX* in *MAT***a** *can1Δ*::*STE2p-spHIS5 lyp1Δ*::*STE3p-LEU2 his3*::*HisG leu2Δ ura3Δ geneX*::*kanMX*) were tested for biofilm formation on polystyrene at 30° in liquid SC with 0.2% glucose and NH_4_^+^ medium. (A) Total biomass (OD_600nm_) *vs.* biofilm formation measured at OD_595nm_ after crystal violet staining. Black circles = all data points for each mutant; red line = correlation between cell density and crystal violet staining; dotted red line = ±2σ. (B) Distribution of normalized biofilm from individual mutants as ln(crystal violet dyed biofilm/total biomass) compared with cell mass. Black circles = all data points for each mutant; red line = correlation between biomass and normalized biofilm; dotted red line = ±2σ. (A and B) Values obtained after 96 hr of biofilm development. (C) Histograms representing median normalized biofilm values of the 4019 deletion mutants assayed in triplicate. Black line = normalized biofilm values for the parental wild-type Σ1278b strain (n = 288) showing Gaussian distribution after 48 hr of growth. Biofilm distribution of the parental strain after 48 hr was used to set cut-off to average ±2σ. Blue bars = biofilms of mutants that formed significantly less biofilm than the parental strain; yellow bars = biofilm values of mutants that formed significantly more biofilm than the wild-type strain. (D) As in (C) after 96 hr of growth. Biofilm distribution of the parental strain after 96 hr of growth was used to set cut-offs to average ±2σ. A complete list of mutants and median normalized biofilm values are listed File S1 and File S2.

The biofilm screen was conducted by growing each of the 4019 mutants in liquid synthetic complete medium with 0.2% glucose and ammonium. Biofilm development was tested in triplicate after 46 hr and 96 hr by staining with crystal violet and removing planktonic cells. Mutants varied greatly in the amount of biomass formed (Figure 2A). To compensate for differences in biomass, values from crystal violet staining were normalized to the total cell mass of planktonic and biofilm-forming cells (OD_600nm_) (Figure 2B). Median normalized biofilm values ln(OD_595nm_/OD_600nm_) were calculated for each mutant at 46 hr and 96 hr (Figure 2, C and D and File S1, File S2). Biofilm formation by the parental strain (n = 288) followed a Gaussian distribution. Thus, the average normalized biofilm value ±2σ for the parental strain was used to identify mutants that formed significantly more or less biofilm than the parental strain (*P* < 0.05). We found that 137 mutants formed significantly less biofilm after 46 hr and, of these, 71 mutants still formed significantly less biofilm after 96 hr (Figure 2, C and D, blue bars, and File S3). A larger set of 427 mutants formed significantly more biofilm after 46 hr (Figure 2C, yellow bars). After 96 hr, 371 mutants formed significantly more biofilm than the parental strain (Figure 2D, yellow bars), with 100 mutants forming more biofilm at both time points (File S3). To obtain a conservative estimate of the genes involved in biofilm development and to avoid growth rate effects, we considered only mutants with significantly altered biofilm effects after both 46 hr and 96 hr. This resulted in 71 candidate genes essential for biofilm development and 100 genes that repressed biofilm development by maintaining a high proportion of planktonic cells (Figure 2, C and D, blue and yellow bars, and File S3).

### Biofilm genes form a large complex regulatory network

**Figure 3:**
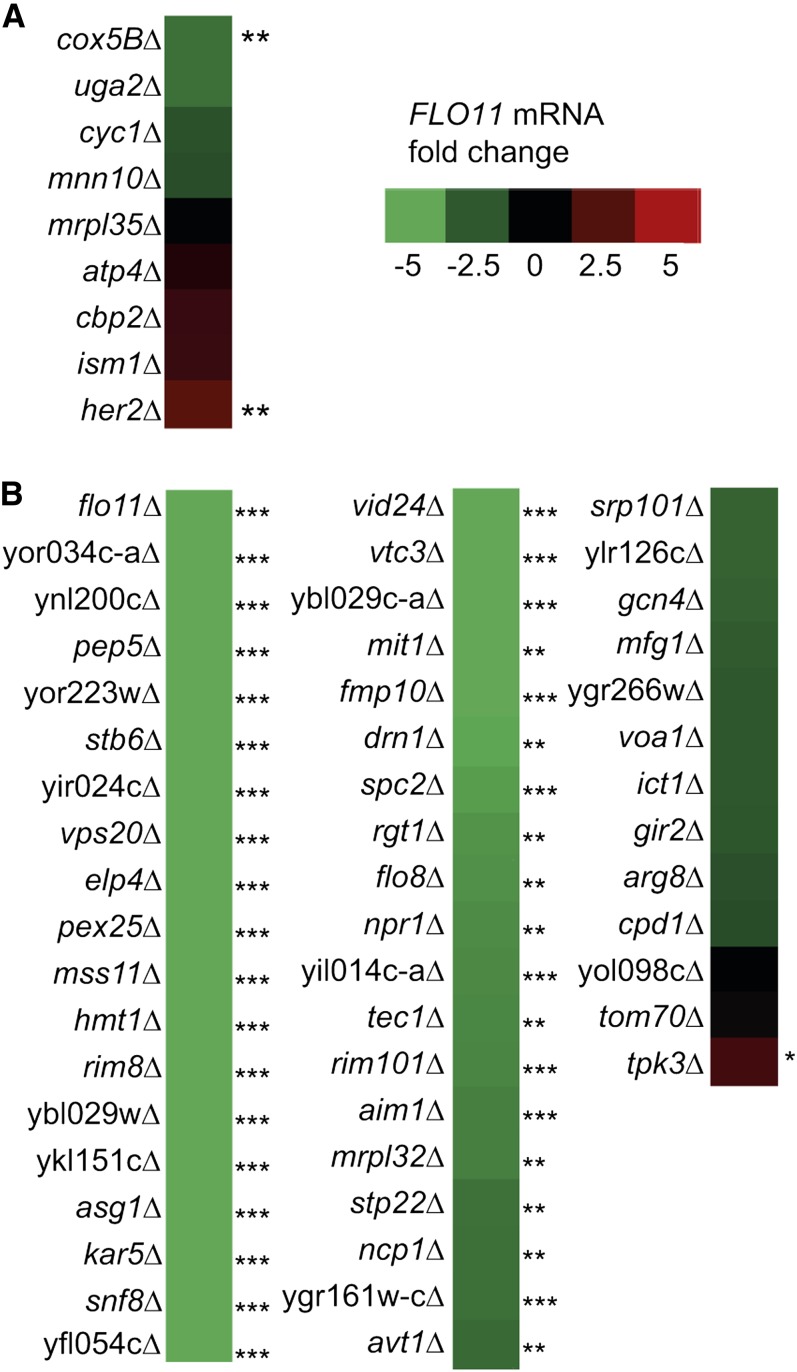
Heat map of *FLO11* mRNA levels in mutants with reduced biofilm. *FLO11* mRNA levels used for the heat map were measured by northern dot blots of mutants grown for 96 hr in 0.2% glucose synthetic medium. *FLO11* mRNA levels were normalized to *ACT1* mRNA levels and the wild-type *ACT1*:*FLO11* mRNA ratio was set as 0. Mutants with significantly altered *FLO11* mRNA levels compared with the parental strain are indicated with asterisks (***P ≤ 0.01, **0.01 < *P* < 0.05, *0.05 < *P* < 0.1; n = 3). (A) Transcript levels of *FLO11* in deletion mutants with increased biofilm formation compared with the parental strain. (B) Transcript levels of *FLO11* in deletion mutants that had lost the ability to form biofilm. Numerical values of *FLO11* mRNA levels are listed in File S5.

To obtain insight into the mechanisms that regulate biofilm development, all 171 biofilm-related genes were grouped into functional categories according to their gene onthology (GO) process (Table 1). Among the genes with a positive effect on biofilm when deleted were several functional groups of genes encoding proteins with mitochondrial function (Table 1 and File S4), and genes encoding respiratory chain components (*P* = 4.56e^−3^) and mitochondrial ribosomal proteins (*P* = 2.19e^−16^) were especially overrepresented. To analyze if the increased proportion of biofilm-forming cells in these mutants was dependent on *FLO11*, we made *FLO11* northern blots of representative mutants with reduced mitochondrial function (Figure 3A and File S5). No change in *FLO11* expression was observed in seven of the tested mutants representing reduced mitochondrial function, suggesting that posttranscriptional mechanisms acted on the *FLO11* gene product, other *FLO* genes were responsible for the increase in biofilm, or mutants with impaired mitochondrial function affected a non-Flo-dependent biofilm mechanism.

**Table 1:** Biofilm gene GO processes for 71 genes that were essential for biofilm development and 100 genes that induced biofilm when deleted (all gene names are given in Table S3)

Mutants Making Significantly Less Biofilm		Mutants Making Significantly More Biofilm	
GO Process Annotation	No. of Mutants	GO Process Annotation	No. of Mutants
Biological process unknown	19	Mitochondrion organization	38
Transcription from RNA polymerase promoter	12	Mitochondrial translation	23
Protein targeting	11	Protein complex biogenesis	13
Response to chemical	7	Cellular respiration	10
Invasive growth in response to glucose limitation	5	Biological process unknown	10
Pseudohyphal growth	5	Cell wall organization or biogenesis	8
Carbohydrate metabolic process	5	Cellular amino acid metabolic process	8
Protein complex biogenesis	5	Sporulation	6
Regulation of transport	4	Transmembrane transport	5
Nucleobase-containing small molecule metabolic process	4	Carbohydrate metabolic process	5
Transmembrane transport	4	DNA replication	4
Mitochondrion organization	4	Mitotic cell cycle	4
Endosomal transport	3	Monocarboxylic acid metabolic process	3
Sporulation	3	Cofactor metabolic process	3
Cell wall organization or biogenesis	3	RNA splicing	3
Signaling	3	Oligosaccharide metabolic process	2
Membrane invagination	2	Membrane invagination	2
Protein maturation	2	Protein glycosylation	2
Organelle fusion	2	Telomere organization	2
Cellular amino acid metabolic process	2	Cytokinesis	2
Lipid metabolic process	2	Cytoplasmic translation	2
Amino acid transport	1	Ion transport	2
Protein folding	1	DNA-templated transcription, initiation	1
Ribosomal large subunit biogenesis	1	Vesicle organization	1
Cellular ion homeostasis	1	Cellular ion homeostasis	1
Cytoplasmic translation	1	Meiotic cell cycle	1
Protein farnesylation	1	Golgi vesicle transport	1
		Nucleobase-containing small molecule metabolic process	1

If two GOs capture identical genes, then only one GO is mentioned (lowest p-value). http://www.yeastgenome.org/cgi-bin/GO/goTermFinder.pl.

Among the 71 genes that were essential for biofilm development were several that were essential for invasive and pseudohyphal growth (Table 1). These included components of the PKA pathway (*FLO8*, *RAS2*, *TPK3*), a MAP kinase pathway (*TEC1*), the Rim pathway (*RIM101*, *RIM8*), and the GCN pathway (*GCN4*), as well as *FLO11* transcription factors that have not been assigned to specific signaling pathways (*MIT1*, *MFG1*, *MSS11*) (Gagiano *et al.* 2003; Ryan *et al.* 2012; Cain *et al.* 2012; Braus *et al.* 2003; Barrales *et al.* 2008; Roberts and Fink 1994; Rupp *et al.* 1999; Robertson and Fink 1998). We also found genes encoding components of the ESCRT complexes that are essential for regulation of *FLO11* (*SNF8*, *STP22*, *VPS36*, *VPS20*, *VPS25*) (Sarode *et al.* 2011). Additionally, the GO revealed a number of genes not previously associated with *FLO11* expression such as *AGT9* and *VTC3*, which are involved in membrane invagination, the vacuolar amino acid transporter *AVT1*, and *NPR1*, a regulator of amino acid transporter endocytosis. Identification of these genes suggested that vacuolar function or transport of one or more proteins to the vacuole was essential for biofilm development. In addition, there is also a group of genes involved in NADH repair such as NADHX epimerase (YNL200C) and NADHX dehydratase (YKL151C). In all, we identified 58 genes not previously associated with biofilm formation or *FLO11* expression (File S3).

Because formation of biofilm in the parental strain was dependent on *FLO11* expression (Figure 1D), we tested the level of *FLO11* mRNA in the mutants that did not form biofilm. Quantitative northern blots for *FLO11* mRNA revealed that 38 mutants had significantly less *FLO11* mRNA than the parental strain (Figure 3B and File S5). As expected, *flo11*, *flo8*, *mit1*, *mss11*, *rim8*, *rim101*, *snf8*, *tec1*, and *vps20* had lower levels of *FLO11* mRNA. A large number of genes not previously implicated in *FLO11* regulation were also found to reduce *FLO11* transcription or mRNA stability when deleted (*AIM1*, *ASG1*, *AVT1*, *DRN1*, *ELP4*, *FMP10*, *HMT1*, *KAR5*, *MRPL32*, *NCP1*, *NPR1*, *PEP5*, *PEX25*, *RGT1*, *SPC2*, *STB6*, *STP22*, *VID24*, *VTC3*, YBL029W, YBL029C-A, YFL054C, YGR161W-C, YIL014C-A, YIR024C, YKL151C, YNL200C, YOR034C-A, YOR223W).

**Figure 4:**
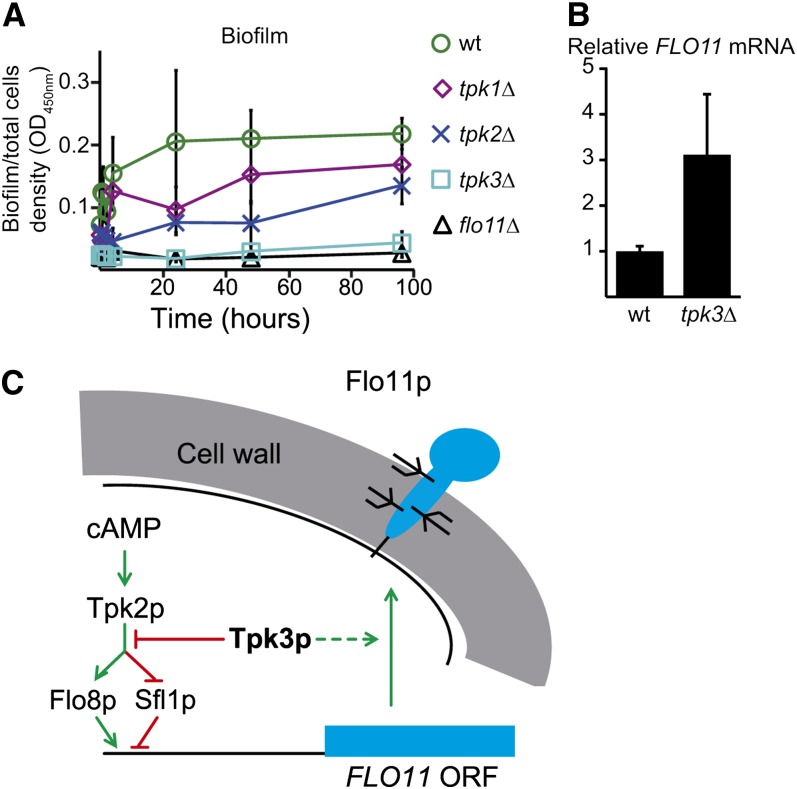
*TPK3* is essential for biofilm development. (A) The percentage of cells forming a biofilm on polystyrene compared with planktonic cells was determined as described in the legend for Figure 1B using OD_450nm_ to determine the biomass of the total population and the biofilm subpopulation. Wild-type = green circle; *flo11* mutant = black triangles; *tpk1* mutant = purple diamond; *tpk2* mutant = blue cross; *tpk3* mutant = turquoise square. (B) *FLO11* mRNA levels in the wild-type and the *tpk3* mutant. Relative *FLO11* mRNA levels were found by normalizing to *ACT1* mRNA levels and the average wild-type *ACT1*:*FLO11* mRNA ratio was set as 1. Both experiments were performed in triplicate (File S5). (C) Model of Tpk3p transcriptional and posttranscriptional regulation of *FLO11*. Green arrows, positive regulation; red bars, negative regulation. Experimental evidence for the interaction between Tpk2p and Tpk3p is also available (Robertson and Fink 1998).

Several factors known to regulate *FLO11* expression did not appear as regulators in the biofilm screen. Most notably, deletion of PKA isoform 2 did not significantly affect biofilm development (Figure 4 and File S1, File S2). The involvement of Tpk2p in *FLO11* transcription and adhesive invasive phenotypes was previously determined by testing on rich complex medium (Robertson and Fink 1998). We found that on liquid synthetic medium, a *tpk2* mutant formed 33%–50% of the amount of biofilm of the wild-type strain (Figure 4A). The Tpk2p homolog Tpk3p, however, was essential for biofilm development (Figure 4A). The positive effect of Tpk3p on biofilm formation was not at the transcriptional level, because the *tpk3* mutant had a 2.7-fold increase in *FLO11* transcript compared with wild-type (Figure 4B and File S5). Hence, Tpk3p appeared to have two roles in *FLO11* regulation: partial repression of *FLO11* transcription but induction of Flo11p expression at a posttranscriptional level. To investigate if Flo11p was expressed in the cell wall of the *tpk3* mutant, we tested the mutant for cell–cell adhesion. The *tpk3* cells grown in liquid synthetic biofilm medium did not adhere to each other as seen for the wild-type strain (data not shown). Hence, the *tpk3* mutant did not appear to express functional Flo11p.

To identify genes involved in regulation of *FLO11* by Tpk3p at the posttranscriptional level, we screened a library of *tpk3 geneX* double mutants for ability to suppress the *tpk3* phenotype and fully restore biofilm formation. Double mutants were made by crossing a biofilm-deficient *tpk3*::*natMX* mutant with the mutant collection using a synthetic genetic array (Tong *et al.* 2001). Diploids were sporulated and haploid double mutants were tested in three independent experiments for ability to form biofilm (Figure S2). The screen identified 35 mutant alleles that suppressed the *tpk3* biofilm phenotype (File S6). However, none of these genes encoded known components of the PKA pathway or components of translation or RNA processing that suggested a pathway or complex through which Tpk3p acted on *FLO11*.

### Genes essential for biofilm formation are also essential for mat formation on semisolid medium

**Figure 5:**
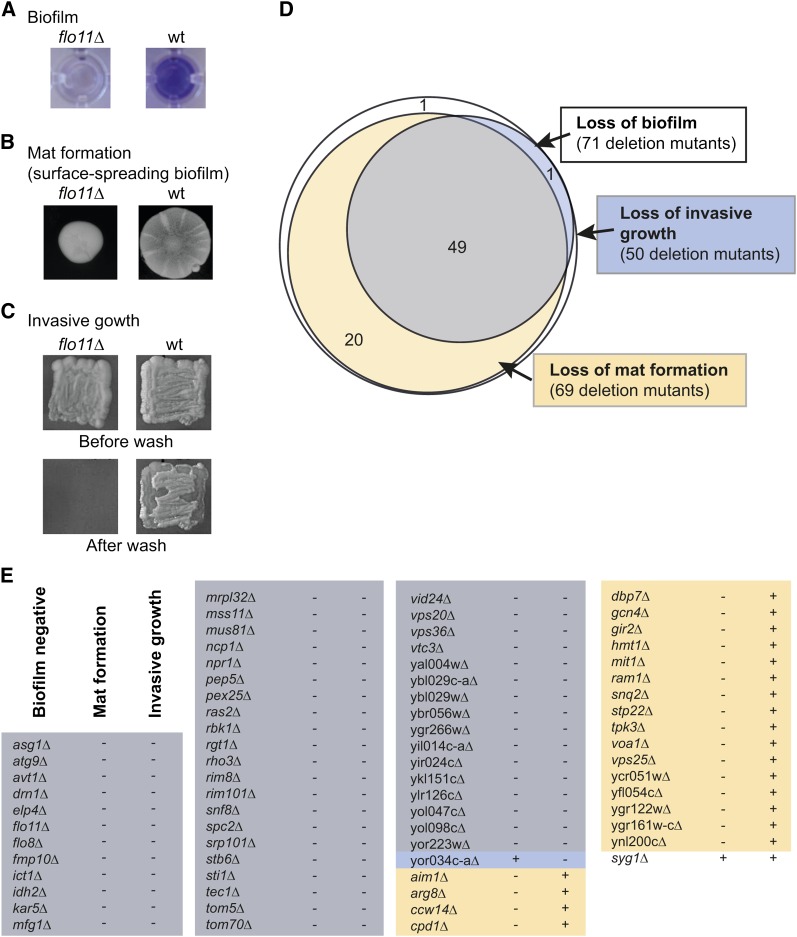
Most genes essential for biofilm are also essential for invasive growth and surface-spreading biofilm formation. (A) Biofilms in microtiter plates stained with crystal violet as described in Figure 2A legend. Color indicates biofilm. Wt, wild-type. Σ1278b parental strain (*MAT***a** *can1Δ*::*STE2p-spHIS5 lyp1Δ*::*STE3p-LEU2 his3*::*HisG leu2Δ ura3Δ*); *flo11Δ*, *flo11*::*kanMX* in the wild-type background. (B) Mat formation of wild-type and lack of mat by *flo11* on semisolid YPD (0.3% agar) after 5 d at room temperature (22°–25°). (C) Invasive growth of wild-type and *flo11* mutant. Invasive growth was tested after 3 d on solid YPD (2% agar) at 30° by washing colonies gently with water to remove nonadhering cells. (D) Diagram of deletion mutants that lost the ability to form biofilm and mats and grow invasively. (E) List of phenotypes of mutants with gene deletion that eliminated biofilm formation. Images of mat formation and invasive growth of all mutants are in Figure S3 and Figure S4.

Next, we tested biofilm-deficient mutants for other *FLO11*-dependent phenotypes (Figure 5, A–C). Mat formation was tested on complex medium with 0.3% agar. Colonies that formed a structured hub and spokes were considered mats, whereas unstructured, smooth colonies were deemed to have lost the ability to form mats (Figure 5B). Of the 71 mutants that lost the ability to form a biofilm in liquid medium on polystyrene, 69 were also unable to form mats, revealing a nearly complete overlap between genes involved in biofilm in synthetic medium and mats formation on semisolid complex medium (Figure 5, D and E and Figure S3). Next, invasive growth was tested on complex medium with 2% agar by growing cells in patches for 2 d and washing the resulting colonies with water (Figure 5C). Colonies that left no macroscopic traces of cells after washing were considered noninvasive, whereas colonies that remained on plates after washing were considered invasive (Figure 5C and Figure S4). Of the 69 mutants that did not form mats or biofilm in synthetic medium, 49 also did not show invasive growth (Figure 5, D and E), revealing a core group of 49 genes essential for the haploid phenotypes of mat and biofilm formation in synthetic medium and invasive growth.

## Discussion

In this work, we found that the *S. cerevisiae* strain Σ1278b showed a stable, dimorphic growth pattern on polystyrene in liquid synthetic complete medium, with both planktonic and biofilm-forming cells. *FLO11* was essential for formation of the biofilm subpopulation. A possible explanation for the dimorphism is that biofilm subpopulations express *FLO11* under control of Flo8p while planktonic populations repress *FLO11* via Sfl1p. It was previously shown that cells in the Σ1278b background show variegated *FLO11* expression that is dependent on Flo8p and Sfl1p (Halme *et al.* 2004). Flo8p and Sfl1p compete for regulation of the *FLO11* promoter and concomitant transcription of either of two ncRNAs in the promoter determines whether the *FLO11* promoter is in a repressed state or a permissive form that allows other transcription factors to induce transcription of *FLO11* (Bumgarner *et al.* 2009). The Sfl1p switch also appeared to be essential for *FLO11*-dependent dimorphism in biofilms, because only a small subpopulation of cells expressed *FLO11* mRNA in the parental strain, whereas this subpopulation increased to 73% in the *sfl1* mutant (Figure 1, F and H).

To investigate the molecular basis for *S. cerevisiae* biofilm formation, we screened a deletion mutant collection in the Σ1278b strain background for loss of biofilm-forming ability. We identified 71 genes with significantly lower biofilm formation than the reference strain (Figure 2, C and D and File S3). Of these, 38 genes resulted in a significant reduction in *FLO11* mRNA levels when deleted, suggesting that biofilm was primarily regulated through transcriptional induction of *FLO11* (Figure 3). A majority of genes have not previously been associated with *FLO11* regulation and the regulatory network underlying *FLO11* thus appears to be much more diverse than previously suggested.

Some of the novel regulators are probably directly involved in *FLO11* regulation while others might have an indirect effect on *FLO11*. For example, Npr1p might be an indirect factor. Npr1p is essential for correct targeting of plasma membrane proteins such as the ammonium permease Mep2p and the general amino acid permease Gap1p (De Craene *et al.* 2001; Vandenbol *et al.* 1990; Lorenz and Heitman 1998). Both Mep2p and Gap1p are involved in *FLO11* expression in some strain backgrounds (Lorenz and Heitman 1998; Torbensen *et al.* 2012). Therefore, the reduction of biofilm formation and expression of *FLO11* in an *npr1* mutant is likely to be a consequence of intracellular retention of Mep2p and/or Gap1p. Although the biofilm assay uncovered several *FLO11* regulators, a number of genes previously described as essential for *FLO11* expression were not found. This was partly the result of a stringent cutoff in the biofilm assay that led to a large number of false negatives. The absence of some known *FLO11* regulators in the biofilm assay could also mean that certain gene products are only conditionally essential for *FLO11* expression. Hence, some genes might be essential for *FLO11*-dependent pseudohyphal growth but not *FLO11*-dependent biofilm development (Ryan *et al.* 2012). One example is the two PKAs, Tpk2p and Tpk3p. Tpk2p is essential for *FLO11* expression in pseudohyphal growth, whereas Tpk3p partially represses *FLO11* transcription under these conditions (Robertson and Fink 1998). We found that Tpk2p was not essential for biofilm formation, although detailed analysis of the *tpk2* mutant revealed that Tpk2p contributed substantially to the biofilm phenotype (Figure 4A). Tpk3p appears to have two effects on biofilm formation. First, a 2.7-fold increase in *FLO11* mRNA in the *tpk3* mutant suggested that Tpk3p repressed *FLO11* transcription in biofilms (Figure 4B). Second, deletion of *TPK3* led to complete biofilm loss (Figure 4A), despite the presence of *FLO11* mRNA in the *tpk3* mutants. Tpk3p thus must also affect posttranscriptional levels of *FLO11*, as indicated in the model in Figure 4C. The positive role of Tpk3p in biofilm development is thus opposite to its role in pseudohyphal growth. Tpk3p is reported to be essential for redistribution of polysomes on glucose starvation (Ashe *et al.* 2000) and might also be involved in polysome maintenance of, for example, polysomes with *FLO11* mRNA. This would explain why the *tpk3* mutant did not form a biofilm despite the high levels of *FLO11* mRNA.

Although a number of genes previously described as regulating *FLO11* were not found in our screen, we did find a strong correlation between genes essential for biofilm formation and genes essential for two other *FLO11*-related phenotypes (Figure 5, D and E). Of the 71 mutants that did not form a biofilm, 69 also lost the ability to form mats, even though the growth conditions for the two phenotypes are very different. Mats are formed at room temperature on semisolid complex medium and are characterized by large, flat colonies with cable-like structures and a central hub. In our experiments, biofilms were formed in liquid synthetic medium at 30°. A smaller subset of 49 genes involved in biofilm and mat formation was also essential for invasive growth on solid complex medium. Although both invasive growth and mat formation are assayed on complex agar medium, the overlap between genes essential for invasive growth and mat formation was smaller than the overlap of genes essential for mat and biofilm formation. This result suggested that a single genetic program mediated the three developmental phenotypes of biofilm and mat formation and invasive growth, whereas at least one other genetic program was specific for mat and biofilm formation (Figure 5, D and E). The common genetic program between the three phenotypes appeared to be almost entirely at the level of *FLO11* transcription. Only a few genes, *ICT1*, *TOM70*, *VOA1*, YGR266W, YLR126C, and YOL098C, did not affect *FLO11* mRNA levels significantly when deleted (Figure 3B).

Several of the genes we identified as involved in biofilm formation were recently reported to be involved in biofilm formation or biofilm-related phenotypes, although their function has not been linked to *FLO11* expression. Ryan *et al.* (2012) found 655 genes essential for mat formation, whereas 211 genes were found to be essential for structured colony morphology (Voordeckers *et al.* 2012). Furthermore, Granek *et al.* (2013) reported that *RGT1* was within a quantitative trait locus found using a biofilm-forming clinical isolate of *S. cerevisiae*. We compared genes essential for biofilm and mat (this study) and genes essential for mat/surface-spreading biofilm identified by Ryan *et al.* (2012) and found an overlap of 38 genes (File S7). Both studies are based on the use of the same deletion strain collection and the *a priori* assumption was an overlap of 69 genes. Some of the discrepancies in our findings might be ascribed to the method by which mutants were recorded as biofilm and mat formers. Although Ryan *et al.* (2012) applied colony size as a measure of mat formation, we used colony morphology to determine if mutants formed mats and the adhering proportion of a population to determine biofilm.

## Conclusion

A genome-wide screen of yeast mutants identified that 71 genes were essential for biofilm development. Half of the genes were required for *FLO11* transcription, but only a small subset is previously described as regulators of *FLO11* transcription. These results revealed that the regulation of biofilm formation and *FLO11* expression is far more complex than previously anticipated. The results of this study will be beneficial for our research in yeast biofilm in general for identification of targets for antifungal drugs and new targets for studying biofilms, mats, and pseudohyphal and invasive growth. Biofilm formed by genetically identical eukaryotic cells might be considered as a primitive form of multicellularity. In this context, the current study therefore provides genetic data for understanding of the development programs for primitive multicellularity in a eukaryotic organism.

## Supplementary Material

Supporting Information

## References

[bib1] Ashe M. P. De Long S. K. Sachs A. B., 2000 Glucose depletion rapidly inhibits translation initiation in yeast. Mol. Biol. Cell 11: 833–848 10712503 10.1091/mbc.11.3.833 PMC14814

[bib2] Barrales R. R. Jimenez J. Ibeas J. I., 2008 Identification of novel activation mechanisms for *FLO11* regulation in *Saccharomyces cerevisiae*. Genetics 178: 145–156 18202364 10.1534/genetics.107.081315 PMC2206066

[bib3] Bayly J. C. Douglas L. M. Pretorius I. S. Bauer F. F. Dranginis A. M., 2005 Characteristics of Flo11-dependent flocculation in *Saccharomyces cerevisiae*. FEMS Yeast Res. 5: 1151–1156 16043420 10.1016/j.femsyr.2005.05.004

[bib4] Braus G. H. Grundmann O. Brückner S. Mösch H. U., 2003 Amino acid starvation and Gcn4p regulate adhesive growth and *FLO11* gene expression in *Saccharomyces cerevisiae*. Mol. Biol. Cell 14: 4272–4284 14517335 10.1091/mbc.E03-01-0042 PMC207018

[bib5] Brückner S. Mösch H. U., 2011 Choosing the right lifestyle: adhesion and development in *Saccharomyces cerevisiae*. FEMS Microbiol. Rev. 36: 25–58 21521246 10.1111/j.1574-6976.2011.00275.x

[bib6] Bumgarner S. L. Dowell R. D. Grisafi P. Gifford D. K. Fink G. R., 2009 Toggle involving cis-interfering noncoding RNAs controls variegated gene expression in yeast. Proc. Natl. Acad. Sci. USA 106: 18321–18326 19805129 10.1073/pnas.0909641106 PMC2775344

[bib7] Bumgarner S. L. Neuert G. Voight B. F. Symbor-Nagrabska A. Grisafi P., 2012 Single-cell analysis reveals that noncoding RNAs contribute to clonal heterogeneity by modulating transcription factor recruitment. Mol. Cell 45: 470–482 22264825 10.1016/j.molcel.2011.11.029 PMC3288511

[bib8] Cain C. W. Lohse M. B. Homann O. R. Sil A. Johnson A. D., 2012 A conserved transcriptional regulator governs fungal morphology in widely diverged species. Genetics 190: 511–521 22095082 10.1534/genetics.111.134080 PMC3276625

[bib9] Chen H. Fink G. R., 2006 Feedback control of morphogenesis in fungi by aromatic alcohols. Genes Dev. 20: 1150–1161 16618799 10.1101/gad.1411806 PMC1472474

[bib10] De Craene J. O. Soetens O. Andre B., 2001 The Npr1 kinase controls biosynthetic and endocytic sorting of the yeast Gap1 permease. J. Biol. Chem. 276: 43939–43948 11500493 10.1074/jbc.M102944200

[bib11] Fichtner L. Schulze F. Braus G. H., 2007 Differential Flo8p-dependent regulation of *FLO1* and *FLO11* for cell-cell and cell-substrate adherence of *S. cerevisiae* S288c. Mol. Microbiol. 66: 1276–1289 18001350 10.1111/j.1365-2958.2007.06014.x PMC2780560

[bib12] Gagiano M. Bester M. Van Dyk D. Franken J. Bauer F. F., 2003 Mss11p is a transcription factor regulating pseudohyphal differentiation, invasive growth and starch metabolism in *Saccharomyces cerevisiae* in response to nutrient availability. Mol. Microbiol. 47: 119–134 12492858 10.1046/j.1365-2958.2003.03247.x

[bib13] Giaever G. Chu A. M. Ni L. Connelly C. Riles L., 2002 Functional profiling of the *Saccharomyces cerevisiae* genome. Nature 418: 387–391 12140549 10.1038/nature00935

[bib14] Gimeno C. J. Ljungdahl P. O. Styles C. A. Fink G. R., 1992 Unipolar cell divisions in the yeast *S. cerevisiae* lead to filamentous growth: regulation by starvation and RAS. Cell 68: 1077–1090 1547504 10.1016/0092-8674(92)90079-r

[bib15] Granek J. A. Murray D. Kayrkci O. Magwene P. M., 2013 The genetic architecture of biofilm formation in a clinical isolate of *Saccharomyces cerevisiae*. Genetics 193: 587–600 23172850 10.1534/genetics.112.142067 PMC3567746

[bib16] Guo B. Styles C. A. Feng Q. Fink G. R., 2000 A *Saccharomyces* gene family involved in invasive growth, cell-cell adhesion, and mating. Proc. Natl. Acad. Sci. USA 97: 12158–12163 11027318 10.1073/pnas.220420397 PMC17311

[bib17] Guthrie C. Fink G. R., 1991 Guide to Yeast Genetics and Molecular Biology. Methods Enzymol, Academic Press, San Diego, CA 2005781

[bib18] Hall-Stoodley L. Costerton J. W. Stoodley P., 2004 Bacterial biofilms: from the natural environment to infectious diseases. Nat. Rev. Microbiol. 2: 95–108 15040259 10.1038/nrmicro821

[bib19] Halme A. Bumgarner S. Styles C. Fink G. R., 2004 Genetic and epigenetic regulation of the *FLO* gene family generates cell-surface variation in yeast. Cell 116: 405–415 15016375 10.1016/s0092-8674(04)00118-7

[bib20] Hawser S. P. Douglas L. J., 1994 Biofilm formation by *Candida* species on the surface of catheter materials in vitro. Infect. Immun. 62: 915–921 8112864 10.1128/iai.62.3.915-921.1994 PMC186203

[bib21] Kuchin S. Vyas V. K. Carlson M., 2002 Snf1 protein kinase and the repressors Nrg1 and Nrg2 regulate *FLO11*, haploid invasive growth, and diploid pseudohyphal differentiation. Mol. Cell. Biol. 22: 3994–4000 12024013 10.1128/MCB.22.12.3994-4000.2002 PMC133850

[bib22] Kuthan M. Devaux F. Janderova B. Slaninova I. Jacq C., 2003 Domestication of wild *Saccharomyces cerevisiae* is accompanied by changes in gene expression and colony morphology. Mol. Microbiol. 47: 745–754 12535073 10.1046/j.1365-2958.2003.03332.x

[bib23] Köhler T. Wesche S. Taheri N. Braus G. H. Mösch H. U., 2002 Dual role of the *Saccharomyces cerevisiae* TEA/ATTS family transcription factor Tec1p in regulation of gene expression and cellular development. Eukaryot. Cell 1: 673–686 12455687 10.1128/EC.1.5.673-686.2002 PMC126755

[bib24] Lamb T. M. Mitchell A. P., 2003 The transcription factor Rim101p governs ion tolerance and cell differentiation by direct repression of the regulatory genes *NRG1* and *SMP1* in *Saccharomyces cerevisiae*. Mol. Cell. Biol. 23: 677–686 12509465 10.1128/MCB.23.2.677-686.2003 PMC151549

[bib25] Liu H. Styles C. A. Fink G. R., 1996 *Saccharomyces cerevisiae* S288C has a mutation in *FLO8*, a gene required for filamentous growth. Genetics 144: 967–978 8913742 10.1093/genetics/144.3.967 PMC1207636

[bib26] Lo W. S. Dranginis A. M., 1996 *FLO11*, a yeast gene related to the *STA* genes, encodes a novel cell surface flocculin. J. Bacteriol. 178: 7144–7151 8955395 10.1128/jb.178.24.7144-7151.1996 PMC178626

[bib27] Lo W. S. Dranginis A. M., 1998 The cell surface flocculin Flo11 is required for pseudohyphae formation and invasion by *Saccharomyces cerevisiae*. Mol. Biol. Cell 9: 161–171 9436998 10.1091/mbc.9.1.161 PMC25236

[bib28] Lorenz M. C. Heitman J., 1998 The MEP2 ammonium permease regulates pseudohyphal differentiation in *Saccharomyces cerevisiae*. EMBO J. 17: 1236–1247 9482721 10.1093/emboj/17.5.1236 PMC1170472

[bib29] Lucchini G. Hinnebusch A. G. Chen C. Fink G. R., 1984 Positive regulatory interactions of the *HIS4* gene of *Saccharomyces cerevisiae*. Mol. Cell. Biol. 4: 1326–1333 6095062 10.1128/mcb.4.7.1326-1333.1984 PMC368915

[bib30] McIsaac R. S. Silverman S. J. Parsons L. Xu P. Briehof R., 2013 Visualization and analysis of mRNA molecules using fluorescence *in situ* hybridization in *Saccharomyces cerevisiae*. J. Vis. Exp. 76: e50382 23793137 10.3791/50382 PMC3727456

[bib31] Nguyen D. Joshi-Datar A. Lepine F. Bauerle E. Olakanmi O., 2011 Active starvation responses mediate antibiotic tolerance in biofilms and nutrient-limited bacteria. Science 334: 982–986 22096200 10.1126/science.1211037 PMC4046891

[bib32] Palkova Z. Janderova B. Gabriel J. Zikanova B. Pospisek M., 1997 Ammonia mediates communication between yeast colonies. Nature 390: 532–536 9394006 10.1038/37398

[bib33] Reynolds T. B. Fink G. R., 2001 Bakers’ yeast, a model for fungal biofilm formation. Science 291: 878–881 11157168 10.1126/science.291.5505.878

[bib34] Roberts R. L. Fink G. R., 1994 Elements of a single MAP kinase cascade in *Saccharomyces cerevisiae* mediate two developmental programs in the same cell type: mating and invasive growth. Genes Dev. 8: 2974–2985 8001818 10.1101/gad.8.24.2974

[bib35] Robertson L. S. Fink G. R., 1998 The three yeast A kinases have specific signaling functions in pseudohyphal growth. Proc. Natl. Acad. Sci. USA 95: 13783–13787 9811878 10.1073/pnas.95.23.13783 PMC24897

[bib36] Rupp S. Summers E. Lo H. J. Madhani H. Fink G., 1999 MAP kinase and cAMP filamentation signaling pathways converge on the unusually large promoter of the yeast *FLO11* gene. EMBO J. 18: 1257–1269 10064592 10.1093/emboj/18.5.1257 PMC1171216

[bib37] Ryan O. Shapiro R. S. Kurat C. F. Mayhew D. Baryshnikova A., 2012 Global gene deletion analysis exploring yeast filamentous growth. Science 337: 1353–1356 22984072 10.1126/science.1224339

[bib38] Sarode N. Miracle B. Peng X. Ryan O. Reynolds T. B., 2011 Vacuolar protein sorting genes regulate mat formation in *Saccharomyces cerevisiae* by Flo11p-dependent and -independent mechanisms. Eukaryot. Cell 10: 1516–1526 21908597 10.1128/EC.05078-11 PMC3209053

[bib39] Smukalla S. Caldara M. Pochet N. Beauvais A. Guadagnini S., 2008 *FLO1* is a variable green beard gene that drives biofilm-like cooperation in budding yeast. Cell 135: 726–737 19013280 10.1016/j.cell.2008.09.037 PMC2703716

[bib40] Tong A. H. Evangelista M. Parsons A. B. Xu H. Bader G. D., 2001 Systematic genetic analysis with ordered arrays of yeast deletion mutants. Science 294: 2364–2368 11743205 10.1126/science.1065810

[bib41] Torbensen R. Møller H. D. Gresham D. Alizadeh S. Ochmann D., 2012 Amino acid transporter genes are essential for *FLO11*-dependent and *FLO11*-independent biofilm formation and invasive growth in *Saccharomyces cerevisiae*. PLoS ONE 7: e41272 22844449 10.1371/journal.pone.0041272 PMC3406018

[bib42] Vachova L. Stovicek V. Hlavacek O. Chernyavskiy O. Stepanek L., 2011 Flo11p, drug efflux pumps, and the extracellular matrix cooperate to form biofilm yeast colonies. J. Cell Biol. 194: 679–687 21875945 10.1083/jcb.201103129 PMC3171128

[bib43] Van De Velde S. Thevelein J. M., 2008 Cyclic AMP-protein kinase A and Snf1 signaling mechanisms underlie the superior potency of sucrose for induction of filamentation in *Saccharomyces cerevisiae*. Eukaryot. Cell 7: 286–293 17890371 10.1128/EC.00276-07 PMC2238163

[bib44] Vandenbol M. Jauniaux J. C. Grenson M., 1990 The *Saccharomyces cerevisiae NPR1* gene required for the activity of ammonia-sensitive amino acid permeases encodes a protein kinase homologue. Mol. Gen. Genet. 222: 393–399 2125693 10.1007/BF00633845

[bib45] Voordeckers K. De Maeyer D. Van Der Zande E. Vinces M. D. Meert W., 2012 Identification of a complex genetic network underlying *Saccharomyces cerevisiae* colony morphology. Mol. Microbiol. 86: 225–239 22882838 10.1111/j.1365-2958.2012.08192.x PMC3470922

[bib46] Xu W. Smith F. J. Jr Subaran R. Mitchell A. P., 2004 Multivesicular body-ESCRT components function in pH response regulation in *Saccharomyces cerevisiae* and *Candida albicans*. Mol. Biol. Cell 15: 5528–5537 15371534 10.1091/mbc.E04-08-0666 PMC532031

