# Supplementary material for: Genetic Basis for Saccharomyces cerevisiae Biofilm in Liquid Medium
Source: G3 (Bethesda). 2014 Jul 9;4(9):1671–80. doi: 10.1534/g3.114.010892 (PMC4169159; doi:10.1534/g3.114.010892)
Supplement: Supporting Information [file supp_4_9_1671__index.html]

Genetic Basis for Saccharomyces cerevisiae Biofilm in Liquid Medium — Supporting Information 

# Genetic Basis for *Saccharomyces cerevisiae* Biofilm in Liquid Medium

## Supporting Information for Andersen *et al.*, 2014

**Files in this Data Supplement:**

- Supporting Information - Figures S1-S4 and Files S1-S7 (PDF, 1 MB)
- Figure S1 - 3D visualization by Confocal Laser Scanning Microscopy (CLSM) on cells grown as described in materials and methods. (PDF, 462 KB)
- Figure S2 - The histogram represents the median of normalized biofilm values of the 4072 *tpk3Δ geneX* double deletion mutants. (PDF, 136 KB)
- Figure S3 - Mat formation assay on semi-solid 0.3% agar YPD. (PDF, 547 KB)
- Figure S4 - Invasive growth assay on solid YPD plates. (PDF, 536 KB)
- File S1 - Biofilm formation of Σ1278b deletion mutant collection after 46 hours. (.zip, 872 KB)
- File S2 - Biofilm formation of Σ1278b deletion mutant collection after 96 hours. (.zip, 911 KB)
- File S3 - Deletion mutants with significant altered biofilm formation after 46 hours and 96 hours. (.zip, 122 KB)
- File S4 - Overrepresented functional categories lost in mutants that form significantly more biofilm http://www.yeastgenome.org/cgi-bin/GO/goTermFinder.pl. (.zip, 120 KB)
- File S5 - Numeric values for data in Figure 3. (.zip, 127 KB)
- File S6 - Mutants suppressing the *tpk3*Δ phenotype. (.zip, 120 KB)
- File S7 - Comparison of biofilm mutants identified in the current study (Figure 2, File S3) and the study of Boone and coworkers (Ryan et al., 2012), where a global analysis of invasive growth and mat formation was done. (.zip, 121 KB)
