# Supplementary material for: Genetic Basis for Saccharomyces cerevisiae Biofilm in Liquid Medium
Source: G3 (Bethesda). 2014 Jul 9;4(9):1671–80. doi: 10.1534/g3.114.010892 (PMC4169159; doi:10.1534/g3.114.010892)
Supplement: Supporting Information [file supp_g3.114.010892_010892SI.pdf]

G3 · Genes | Genomes | Genetics

Genetic basis for *Saccharomyces cerevisiae* biofilm in liquid medium

Kaj Scherz Andersen*
Email: Kajan@bio.ku.dk,

Rasmus Bojsen §[1]
Email: rakb@bio.dtu.dk

Laura Gro Rejkjær Sørensen*
Email: Laura.gro.90@gmail.com

Martin Weiss Nielsen §[1]
Email: mawen@vet.dtu.dk

Michael Lisby*
Email: mlisby@bio.ku.dk

Anders Folkesson§[1]
Email: afol@vet.dtu.dk

Birgitte Regenberg*
Email: bregenberg@bio.ku.dk

*Department of Biology, University of Copenhagen, Copenhagen, DK. §Department of Systems Biology, Technical University of Denmark, Copenhagen, DK.
1) Present address: National Veterinary Institute, Technical University of Denmark, Copenhagen, DK.
#Address correspondence to Birgitte Regenberg, bregenberg@bio.ku.dk. Department of Biology, University of Copenhagen, Universitetsparken 13, DK-2100 Copenhagen, phone: (+45)35321680.

DOI: 10.1534/g3.114.010892

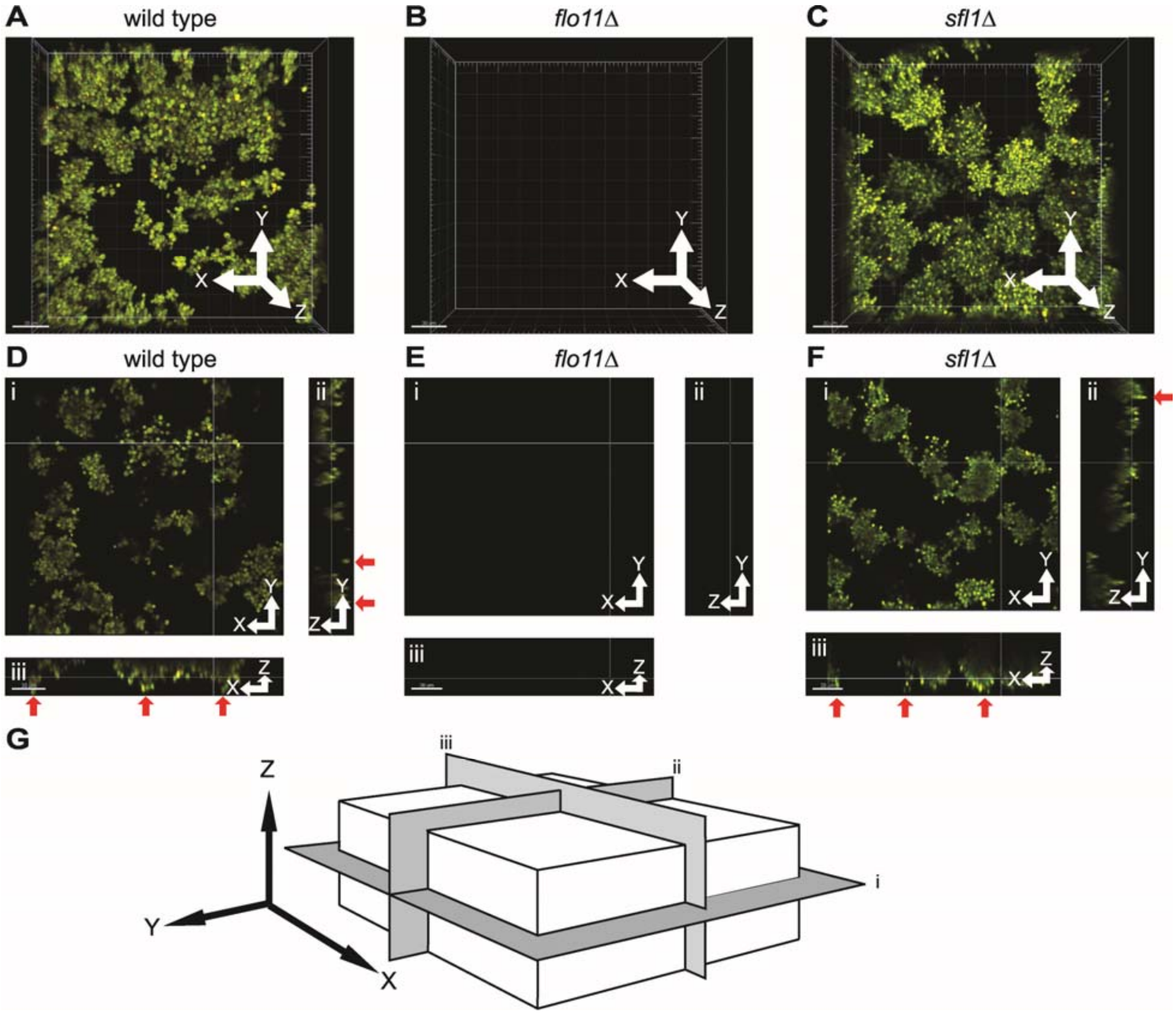

Figure S1 3D visualization by Confocal Laser Scanning Microscopy (CLSM) on cells grown as described in materials and methods. **A**, **B** and **C** are 3D reconstructions of biofilm made from 2-µm thick images in stacks of up to 75 individual images. **D**, **E** and **F** are sections in the X–Y, X–Z and Y–Z dimensions of the biofilm. Red arrows indicate cell-surface attachment point. **G**) Schematic drawing of cross section through biofilm (white box) in the X-Y dimension (i), Y-Z dimension (ii) and X-Z dimension (iii). Both reconstruction images and sections through the biofilm were made with IMARIS software (Bitplane) from raw CLSM images. CLSM was performed with a Zeiss LSM510 microscope using a 639/0.95NA water immersion lens. Bar 30 µm.

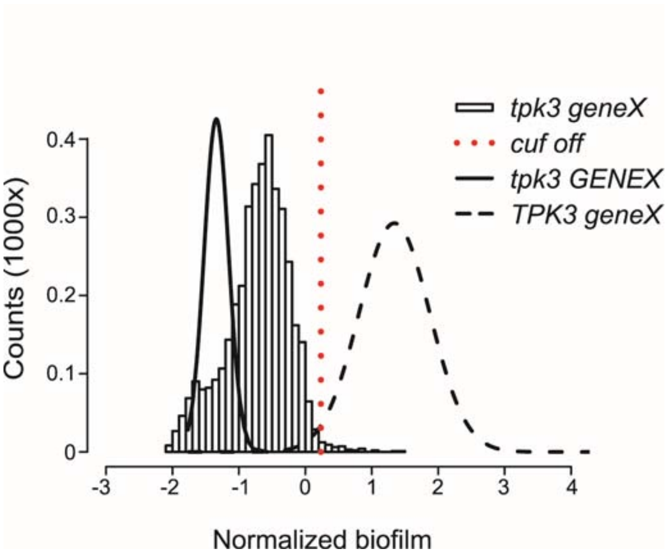

Figure S2 The histogram represents the median of normalized biofilm values of the 4072 *tpk3*Δ *geneX* double deletion mutants. Solid black line indicates a Gaussian distribution of normalized biofilm of the *tpk3*Δ strain (n=192). Dashed black line indicates the Gaussian distribution of normalized of *TPK3* biofilm. Red dotted line represents the threshold of "normal" biofilm formation (-2σ cut-off of the distribution for the *TPK3* parental strain).

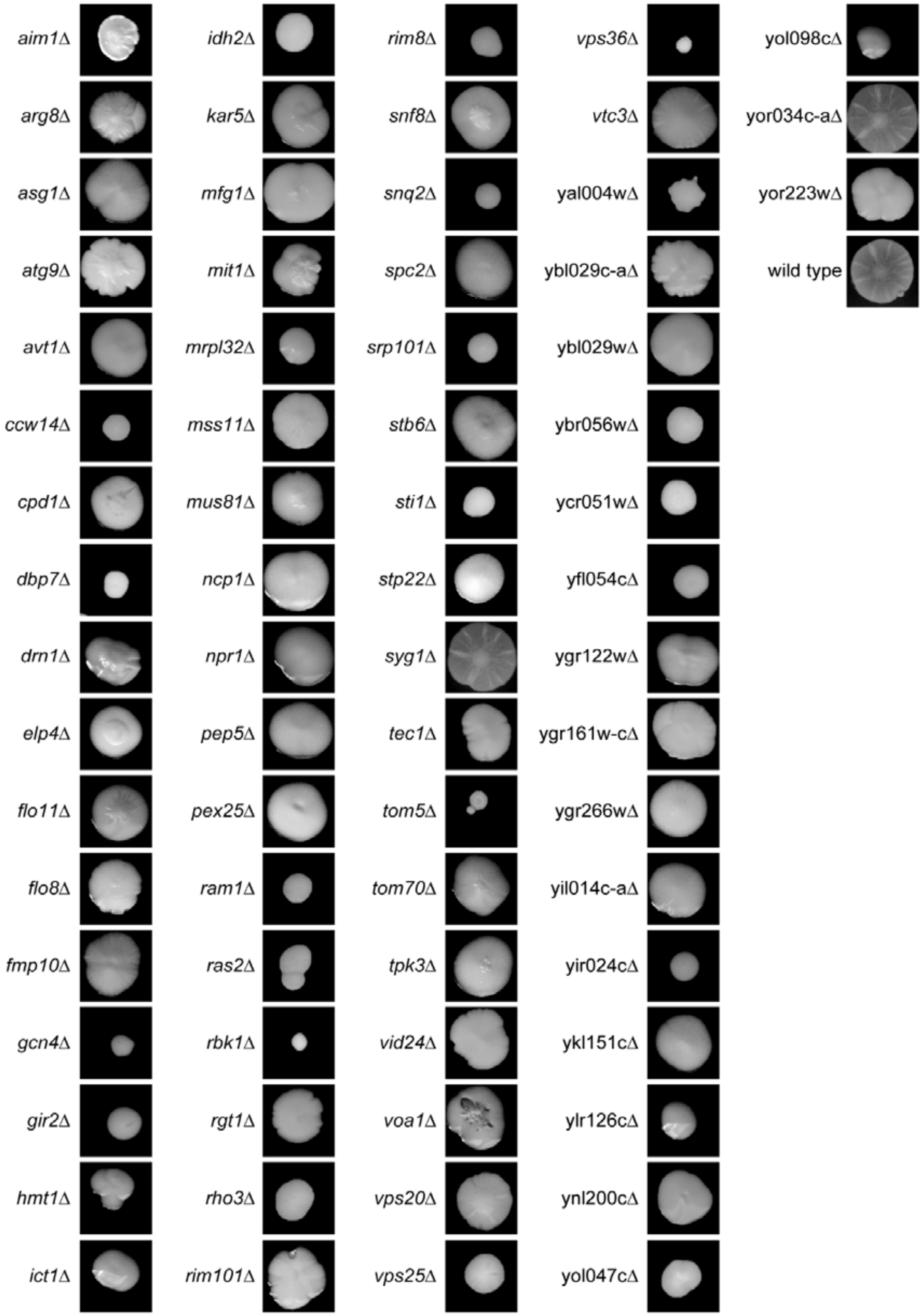

Figure S3 Mat formation assay on semi-solid 0.3% agar YPD. Colonies were grown for 5 days, room temperature, as described in materials and methods. The parental Σ1278b shows the classical hub and spoke structure. Only mutants that had lost the ability to form biofilm were assayed.

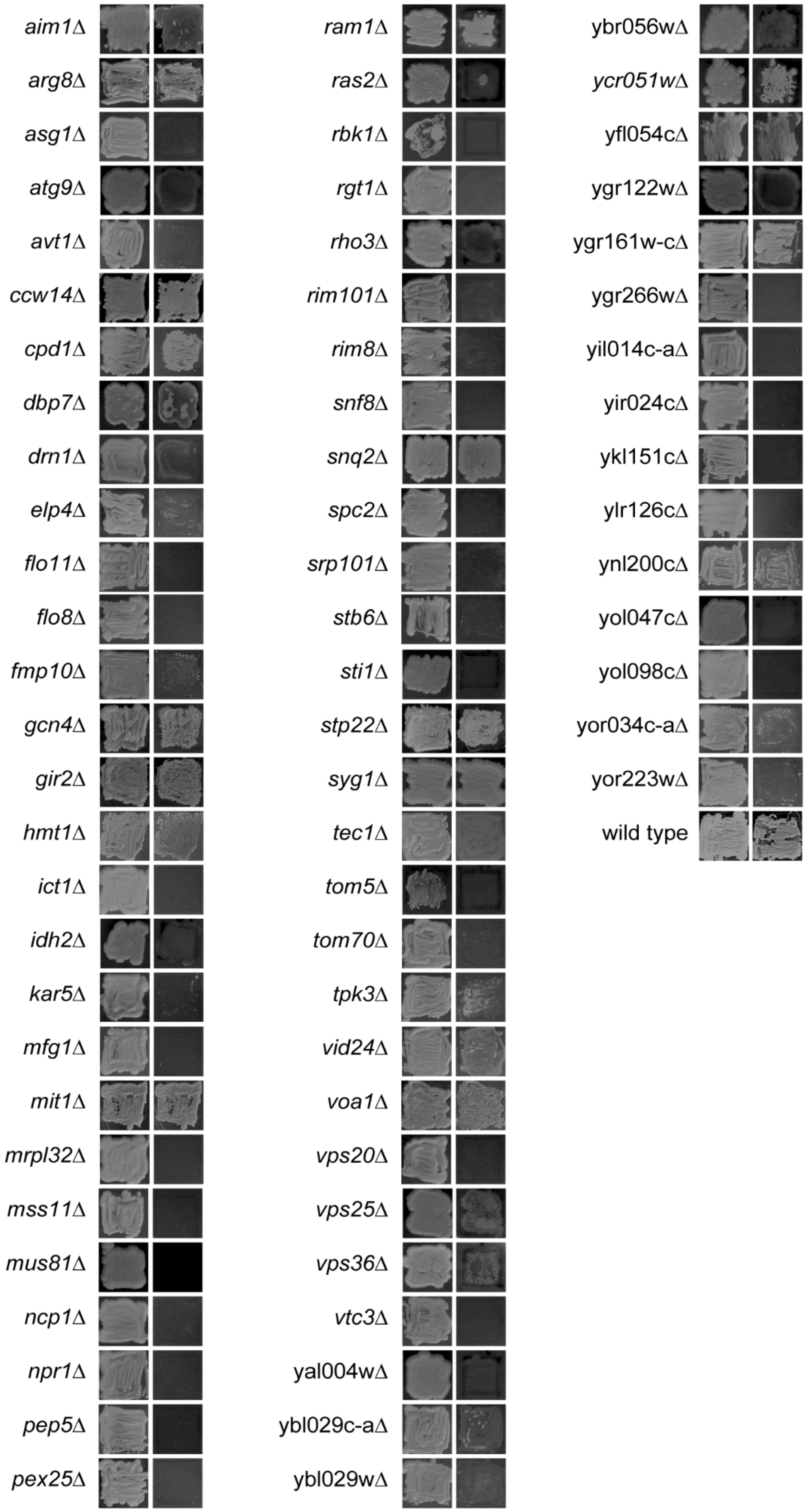

Figure S4 Invasive growth assay on solid YPD plates. Mutants were in patches and tested for invasive growth as described in materials and methods. Each deletion mutant is depictured with an image before and after wash. Only mutants that had lost the ability to form biofilm were assayed.

Files S1-S7

Available for download as Excel files at http://www.g3journal.org/lookup/suppl/doi:10.1534/g3.114.010892/-/DC1

File S1 Biofilm formation of Σ1278b deletion mutant collection after 46 hours. First column; the corresponding ORF name. Second column; gene deleted in the mutant. Third column; Replicate number. Fourth column; Biomass measured with OD_{600nm}. Fifth column; Biofilm stained with crystal violet measured at OD_{595nm}. Sixth column; Normalized biofilm (Crystal violet stained biofilm (OD_{595nm})/Biomass (OD_{600nm})). Seventh column; median from three replicates of normalized biofilm score. Eighth column; ln(median of normalized biofilm). Biofilm was scored by normalizing each value of crystal violet stained biofilm with the total biomass of each sample and log transforming the value, ln(OD_{595nm}/OD_{600nm}). The normalized biofilm scores were subsequently used to determine the median biofilm-score value for each mutant. NA: Data not available.

File S2 Biofilm formation of Σ1278b deletion mutant collection after 96 hours. First column; the corresponding ORF name. Second column; gene deleted in the mutant. Third column; Replicate number. Fourth column; Biomass measured with OD_{600nm}. Fifth column; Biofilm stained with crystal violet measured at OD_{595nm}. Sixth column; Normalized biofilm (Crystal violet stained biofilm (OD_{595nm})/Biomass (OD_{600nm})). Seventh column; median from three replicates of normalized biofilm score. Eighth column; ln(median of normalized biofilm). Biofilm was scored by normalizing each value of crystal violet stained biofilm with the total biomass of each sample and log transforming the value, ln(OD_{595nm}/OD_{600nm}). The normalized biofilm scores were subsequently used to determine the median biofilm-score value for each mutant. NA: Data not available.

File S3 Deletion mutants with significant altered biofilm formation after 46 hours and 96 hours. First column, ORF deleted in mutants forming significantly less biofilm; second column, corresponding gene deleted in mutants forming significantly less biofilm. Third column, ORF deleted in mutants forming significantly more biofilm; forth column, corresponding gene deleted in mutants forming significantly more biofilm.

File S4 Overrepresented functional categories lost in mutants that form significantly more biofilm http://www.yeastgenome.org/cgi-bin/GO/goTermFinder.pl. For all listed functional categories, a representative mutant was chosen (third column) and the *FLO11* mRNA levels measured with Northern blot (Figure 3A).

File S5 Numeric values for data in Figure 3. *FLO11/ACT1* mRNA levels were determined in three independent experiments. In a few cases data are not available (NA). First column, ORF deleted in mutants; second column, corresponding gene deleted in mutants; third, fourth, fifth, and sixth column, relative *FLO11/ACT1* mRNA ratios normalized to wild type *FLO11/ACT1* mRNA ratios; Seventh column, average of third, fourth, fifth, and sixth column; Eight column, standard deviation of average relative *FLO11/ACT1* mRNA ratios normalized to wild type *FLO11/ACT1* mRNA ratios; Ninth column, Average fold change of *FLO11/ACT1* mRNA ratios normalized to wild type *FLO11/ACT1* mRNA ratios; Tenth column, p values of Student's t test from at least 3 experiments; Eleventh column, Significance in change of *FLO11*/*ACT1* mRNA in deletion mutant compared to wild type *FLO11*/*ACT1* mRNA. (***$p \leq 0.01$, ** $0.01 < p < 0.05$, * $0.05 < p < 0.1$, n=3).

File S6 Mutants suppressing the *tpk3*Δ phenotype. First column; genotype of the *tpk3Δ* suppressor mutants. Second column; median of normalized biofilm values, calculated as described in materials and methods. Data are based on three independent experiments.

File S7 Comparison of biofilm mutants identified in the current study (Figure 2, File S3) and the study of Boone and coworkers (Ryan et al., 2012), where a global analysis of invasive growth and mat formation was done. First column, ORF deleted in mutants; second column, corresponding gene deleted in mutants; third column, Mat phenotype recorded in this study; forth column, Invasive phenotype recorded in this study; fifth column, Mat phenotype recorded by (Ryan et al., 2012); Sixth column invasive phenotype recorded by (Ryan et al., 2012).
