# Supplementary material for: Genetic Basis for Saccharomyces cerevisiae Biofilm in Liquid Medium
Source: G3 (Bethesda). 2014 Jul 9;4(9):1671–80. doi: 10.1534/g3.114.010892 (PMC4169159; doi:10.1534/g3.114.010892)
Supplement: Supporting Information [file supp_g3.114.010892_FigureS1.pdf]

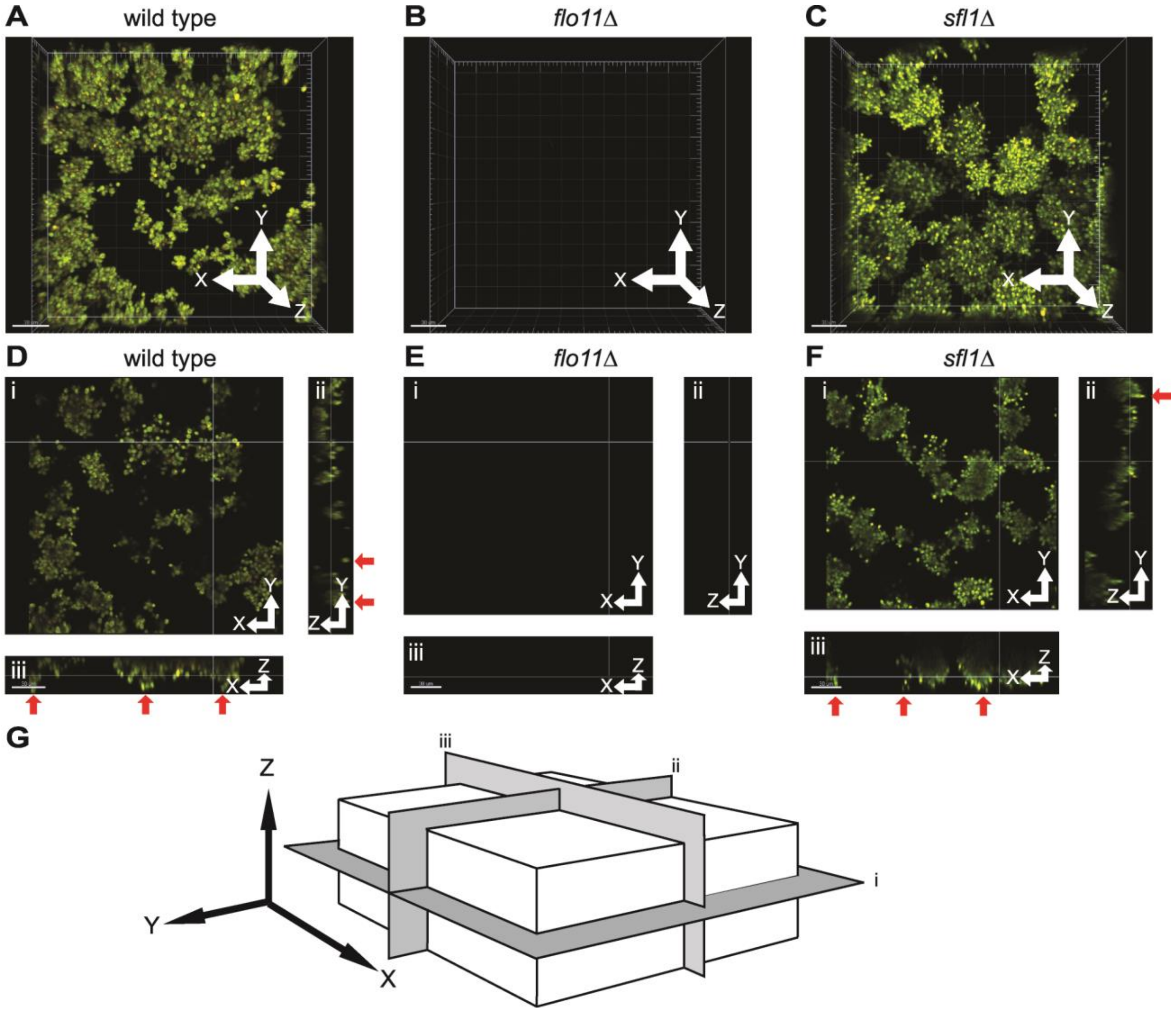


**Figure S1** 3D visualization by Confocal Laser Scanning Microscopy (CLSM) on cells grown as described in materials and methods. **A**, **B** and **C** are 3D reconstructions of biofilm made from 2-µm thick images in stacks of up to 75 individual images. **D**, **E** and **F** are sections in the X–Y, X–Z and Y–Z dimensions of the biofilm. Red arrows indicate cell-surface attachment point. **G**) Schematic drawing of cross section through biofilm (white box) in the X-Y dimension (i), Y-Z dimension (ii) and X-Z dimension (iii). Both reconstruction images and sections through the biofilm were made with IMARIS software (Bitplane) from raw CLSM images. CLSM was performed with a Zeiss LSM510 microscope using a 639/0.95NA water immersion lens. Bar 30 µm.
