# Supplementary material for: Genetic Basis for Saccharomyces cerevisiae Biofilm in Liquid Medium
Source: G3 (Bethesda). 2014 Jul 9;4(9):1671–80. doi: 10.1534/g3.114.010892 (PMC4169159; doi:10.1534/g3.114.010892)
Supplement: Supporting Information [file supp_g3.114.010892_FigureS2.pdf]

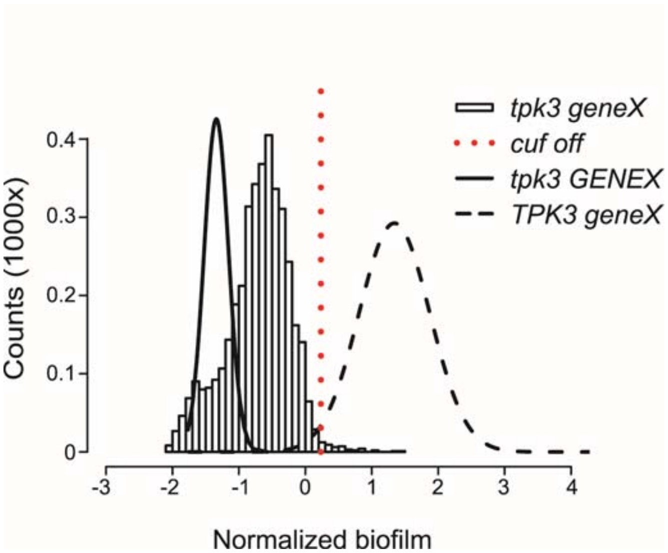


**Figure S2** The histogram represents the median of normalized biofilm values of the 4072 *tpk3*Δ *geneX* double deletion mutants. Solid black line indicates a Gaussian distribution of normalized biofilm of the *tpk3*Δ strain (n=192). Dashed black line indicates the Gaussian distribution of normalized of *TPK3* biofilm. Red dotted line represents the threshold of "normal" biofilm formation (-2σ cut-off of the distribution for the *TPK3* parental strain).
