# Supplementary material for: Genetic Basis for Saccharomyces cerevisiae Biofilm in Liquid Medium
Source: G3 (Bethesda). 2014 Jul 9;4(9):1671–80. doi: 10.1534/g3.114.010892 (PMC4169159; doi:10.1534/g3.114.010892)
Supplement: Supporting Information [file supp_g3.114.010892_FigureS3.pdf]

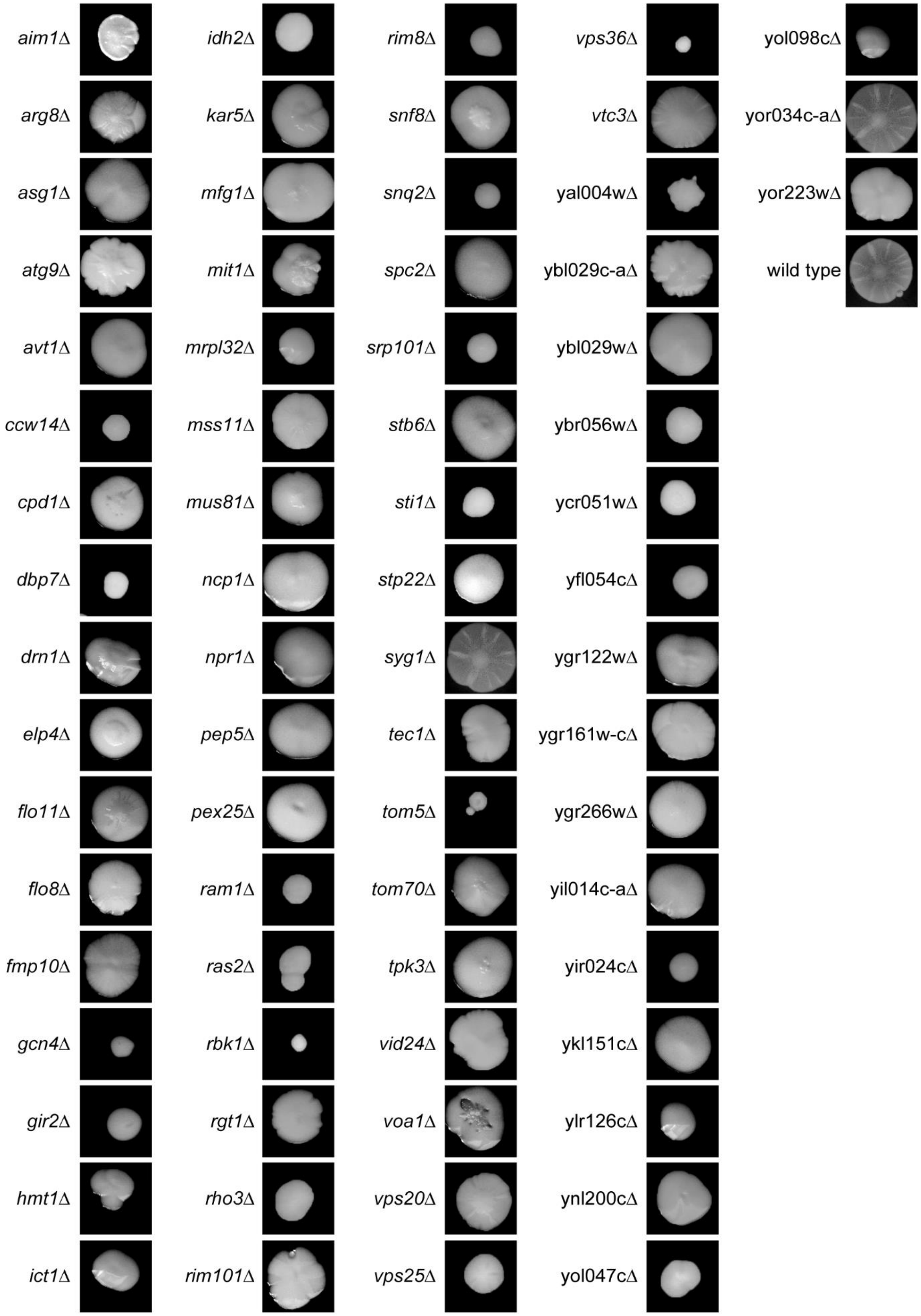

Figure S3 Mat formation assay on semi-solid 0.3% agar YPD. Colonies were grown for 5 days, room temperature, as described in materials and methods. The parental Σ1278b shows the classical hub and spoke structure. Only mutants that had lost the ability to form biofilm were assayed.
