# Supplementary material for: Genetic Basis for Saccharomyces cerevisiae Biofilm in Liquid Medium
Source: G3 (Bethesda). 2014 Jul 9;4(9):1671–80. doi: 10.1534/g3.114.010892 (PMC4169159; doi:10.1534/g3.114.010892)
Supplement: Supporting Information [file supp_g3.114.010892_FigureS4.pdf]

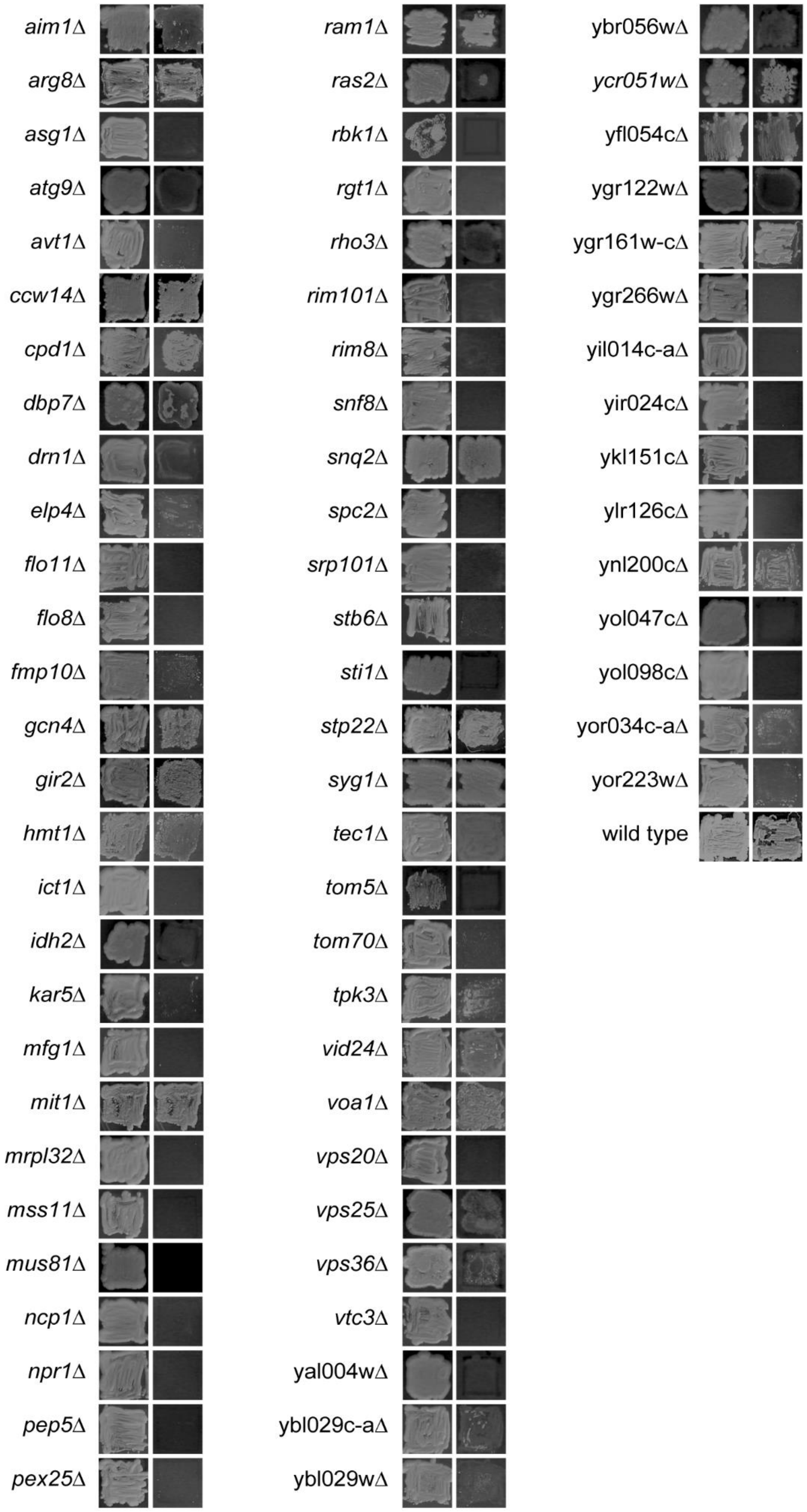

Figure S4 Invasive growth assay on solid YPD plates. Mutants were in patches and tested for invasive growth as described in materials and methods. Each deletion mutant is depictured with an image before and after wash. Only mutants that had lost the ability to form biofilm were assayed.
