# Supplementary material for: Genetic Basis for Saccharomyces cerevisiae Biofilm in Liquid Medium
Source: G3 (Bethesda). 2014 Jul 9;4(9):1671–80. doi: 10.1534/g3.114.010892 (PMC4169159; doi:10.1534/g3.114.010892)
Supplement: Supporting Information [file supp_g3.114.010892_FileS1.zip › FileS1/READ_ME.pdf]

File S1 Biofilm formation of Σ1278b deletion mutant collection after 46 hours. First column; the corresponding ORF name. Second column; gene deleted in the mutant. Third column; Replicate number. Fourth column; Biomass measured with OD_{600nm}. Fifth column; Biofilm stained with crystal violet measured at OD_{595nm}. Sixth column; Normalized biofilm (Crystal violet stained biofilm (OD_{595nm})/Biomass (OD_{600nm})). Seventh column; median from three replicates of normalized biofilm score. Eighth column; ln(median of normalized biofilm). Biofilm was scored by normalizing each value of crystal violet stained biofilm with the total biomass of each sample and log transforming the value, ln(OD_{595nm}/OD_{600nm}). The normalized biofilm scores were subsequently used to determine the median biofilm-score value for each mutant. NA: Data not available.
