# Supplementary material for: Genetic Basis for Saccharomyces cerevisiae Biofilm in Liquid Medium
Source: G3 (Bethesda). 2014 Jul 9;4(9):1671–80. doi: 10.1534/g3.114.010892 (PMC4169159; doi:10.1534/g3.114.010892)
Supplement: Supporting Information [file supp_g3.114.010892_FileS3.zip › FileS3/READ_ME.pdf]

File S3 Deletion mutants with significant altered biofilm formation after 46 hours and 96 hours. First column, ORF deleted in mutants forming significantly less biofilm; second column, corresponding gene deleted in mutants forming significantly less biofilm. Third column, ORF deleted in mutants forming significantly more biofilm; forth column, corresponding gene deleted in mutants forming significantly more biofilm.
