# Supplementary material for: Genetic Basis for Saccharomyces cerevisiae Biofilm in Liquid Medium
Source: G3 (Bethesda). 2014 Jul 9;4(9):1671–80. doi: 10.1534/g3.114.010892 (PMC4169159; doi:10.1534/g3.114.010892)
Supplement: Supporting Information [file supp_g3.114.010892_FileS4.zip › FileS4/READ_ME.pdf]

File S4 Overrepresented functional categories lost in mutants that form significantly more biofilm http://www.yeastgenome.org/cgi-bin/GO/goTermFinder.pl. For all listed functional categories, a representative mutant was chosen (third column) and the *FLO11* mRNA levels measured with Northern blot (Figure 3A).
