# Supplementary material for: Genetic Basis for Saccharomyces cerevisiae Biofilm in Liquid Medium
Source: G3 (Bethesda). 2014 Jul 9;4(9):1671–80. doi: 10.1534/g3.114.010892 (PMC4169159; doi:10.1534/g3.114.010892)
Supplement: Supporting Information [file supp_g3.114.010892_FileS5.zip › FileS5/READ_ME.pdf]

File S5 Numeric values for data in Figure 3. *FLO11/ACT1* mRNA levels were determined in three independent experiments. In a few cases data are not available (NA). First column, ORF deleted in mutants; second column, corresponding gene deleted in mutants; third, fourth, fifth, and sixth column, relative *FLO11/ACT1* mRNA ratios normalized to wild type *FLO11/ACT1* mRNA ratios; Seventh column, average of third, fourth, fifth, and sixth column; Eight column, standard deviation of average relative *FLO11/ACT1* mRNA ratios normalized to wild type *FLO11/ACT1* mRNA ratios; Ninth column, Average fold change of *FLO11/ACT1* mRNA ratios normalized to wild type *FLO11/ACT1* mRNA ratios; Tenth column, *p* values of Student's *t* test from at least 3 experiments; Eleventh column, Significance in change of *FLO11*/*ACT1* mRNA in deletion mutant compared to wild type *FLO11*/*ACT1* mRNA. (***$p \leq 0.01$, ** $0.01 < p < 0.05$, * $0.05 < p < 0.1$, n=3).
