# Supplementary material for: Genetic Basis for Saccharomyces cerevisiae Biofilm in Liquid Medium
Source: G3 (Bethesda). 2014 Jul 9;4(9):1671–80. doi: 10.1534/g3.114.010892 (PMC4169159; doi:10.1534/g3.114.010892)
Supplement: Supporting Information [file supp_g3.114.010892_FileS6.zip › FileS6/READ_ME.pdf]

File S6 Mutants suppressing the *tpk3*Δ phenotype. First column; genotype of the *tpk3Δ* suppressor mutants. Second column; median of normalized biofilm values, calculated as described in materials and methods. Data are based on three independent experiments.
