# Supplementary material for: Genetic Basis for Saccharomyces cerevisiae Biofilm in Liquid Medium
Source: G3 (Bethesda). 2014 Jul 9;4(9):1671–80. doi: 10.1534/g3.114.010892 (PMC4169159; doi:10.1534/g3.114.010892)
Supplement: Supporting Information [file supp_g3.114.010892_FileS7.zip › FileS7/READ_ME.pdf]

File S7 Comparison of biofilm mutants identified in the current study (Figure 2, File S3) and the study of Boone and coworkers (Ryan et al., 2012), where a global analysis of invasive growth and mat formation was done. First column, ORF deleted in mutants; second column, corresponding gene deleted in mutants; third column, Mat phenotype recorded in this study; forth column, Invasive phenotype recorded in this study; fifth column, Mat phenotype recorded by (Ryan et al., 2012); Sixth column invasive phenotype recorded by (Ryan et al., 2012).
